# Identification of β-Lactams Active against *Mycobacterium tuberculosis* by a Consortium of Pharmaceutical Companies and Academic Institutions

**DOI:** 10.1021/acsinfecdis.1c00570

**Published:** 2022-02-22

**Authors:** Ben Gold, Jun Zhang, Landys Lopez Quezada, Julia Roberts, Yan Ling, Madeleine Wood, Wasima Shinwari, Laurent Goullieux, Christine Roubert, Laurent Fraisse, Eric Bacqué, Sophie Lagrange, Bruno Filoche-Rommé, Michal Vieth, Philip A. Hipskind, Louis N. Jungheim, Jeffrey Aubé, Sarah M. Scarry, Stacey L. McDonald, Kelin Li, Andrew Perkowski, Quyen Nguyen, Véronique Dartois, Matthew Zimmerman, David B. Olsen, Katherine Young, Shilah Bonnett, Douglas Joerss, Tanya Parish, Helena I. Boshoff, Kriti Arora, Clifton E. Barry, Laura Guijarro, Sara Anca, Joaquín Rullas, Beatriz Rodríguez-Salguero, Maria S. Martínez-Martínez, Esther Porras-De Francisco, Monica Cacho, David Barros-Aguirre, Paul Smith, Steven J. Berthel, Carl Nathan, Robert H. Bates

**Affiliations:** † Department of Microbiology & Immunology, Weill Cornell Medicine, 413 East 69th Street, New York, New York 10021, United States; ‡ Sanofi, Infectious Diseases Therapeutic Area, 69280 Marcy l’Étoile, France; $$ Sanofi, Integrated Drug Discovery, 94400 Vitry sur Seine, France; § Evotec (Lyon) SAS, 69007 Lyon, France; ∥ Lilly Biotechnology Center, Eli Lilly and Company, 10290 Campus Point Dr, San Diego, California 92121, United States; ⊥ Lilly Research Laboratories, Lilly Corporate Center, Eli Lilly and Company, Indianapolis, Indiana 46285, United States; # YourEncore, 20 North Meridian Street, Indianapolis, Indiana 46204, United States; ¶ Division of Chemical Biology and Medicinal Chemistry, UNC Eshelman School of Pharmacy, University of North Carolina, Chapel Hill, North Carolina 27599, United States; ∇ Public Health Research Institute, New Jersey Medical School, Rutgers, The State University of New Jersey, Newark, New Jersey 07103, United States; ○ Merck & Co., Inc., Infectious Diseases, 770 Sumneytown Pike, West Point, Pennsylvania 19486, United States; ⧫ TB Discovery Research, Infectious Disease Research Institute, 1616 Eastlake Ave E, Suite 400, Seattle, Washington 98102, United States; †† Tuberculosis Research Section, Laboratory of Clinical Immunology and Microbiology, Bethesda, Maryland 20892, United States; ‡‡ Global Health Pharma R&D, GlaxoSmithKline, Severo Ochoa 2, Tres Cantos, Madrid 28760, Spain; §§ Independent Consultant, Global Health Pharma R&D, GlaxoSmithKline, Severo Ochoa 2, Tres Cantos, Madrid 28760, Spain; ∥∥ Panorama Global, 2101 4th Avenue, Suite 2100, Seattle, Washington 98121, United States

**Keywords:** Mycobacterium tuberculosis, tuberculosis, β-lactam, clavulanate, high-throughput screening, consortium

## Abstract

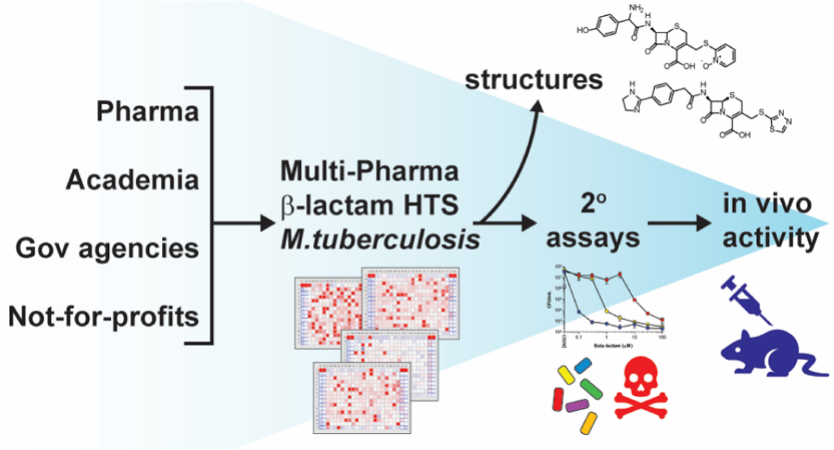

Rising antimicrobial resistance challenges our ability to combat bacterial infections. The problem is acute for tuberculosis (TB), the leading cause of death from infection before COVID-19. Here, we developed a framework for multiple pharmaceutical companies to share proprietary information and compounds with multiple laboratories in the academic and government sectors for a broad examination of the ability of β-lactams to kill *Mycobacterium tuberculosis* (Mtb). In the TB Drug Accelerator (TBDA), a consortium organized by the Bill & Melinda Gates Foundation, individual pharmaceutical companies collaborate with academic screening laboratories. We developed a higher order consortium within the TBDA in which four pharmaceutical companies (GlaxoSmithKline, Sanofi, MSD, and Lilly) collectively collaborated with screeners at Weill Cornell Medicine, the Infectious Disease Research Institute (IDRI), and the National Institute of Allergy and Infectious Diseases (NIAID), pharmacologists at Rutgers University, and medicinal chemists at the University of North Carolina to screen ∼8900 β-lactams, predominantly cephalosporins, and characterize active compounds. In a striking contrast to historical expectation, 18% of β-lactams screened were active against Mtb, many without a β-lactamase inhibitor. One potent cephaloporin was active in Mtb-infected mice. The steps outlined here can serve as a blueprint for multiparty, intra- and intersector collaboration in the development of anti-infective agents.

The economic disincentives that discourage antibiotic research and development are deepening the global crisis of antimicrobial resistance (AMR).^1^ The world needs novel, safe, and effective drugs against pathogens resistant to existing drugs and demonstrably noninferior to existing agents in patients with drug-sensitive infections.^2,3^ However, without access to the compound collections, skill sets, historical experience, and financial resources of large drug companies, it is difficult for academic labs or small firms to develop new antimicrobials and nearly impossible to bring them to the market.^4^

Penicillin became widely available through the efforts of a consortium of chemistry companies and academics organized by the US Government during World War II.^5^ The postwar market for antibacterials proved so fruitful that the participating chemistry companies became drug companies, entered into mutual competition, and inspired other firms to do the same. In the post-World War II environment, pharma–pharma and pharma–academic consortium-based drug discovery was rare until 2010,^5−9^ when the Bill & Melinda Gates Foundation launched the Tuberculosis Drug Accelerator (TBDA).^5,10^ The TBDA is a collaborative framework for multiple commercial, not-for-profit, and academic entities to share ideas, technologies, compounds, assays, risks, and rewards. Here, we report a drug discovery effort hosted within the TBDA that involved an even greater degree of collaboration. This report describes the details of its structure and the fruits of its efforts in TB research in hope that this model may be broadly useful to attack AMR or other diseases.

*Mycobacterium tuberculosis* (Mtb) caused an estimated 1.4 million deaths in 2019, making tuberculosis (TB) the single leading cause of death from an infectious disease at that time.^11^ Resistance is rising to all drugs approved for use in the treatment of TB, such that Mtb is among the numerically leading causes of drug-resistant bacterial infections. Recently, new TB drug candidates have entered the pipeline.^12−14^ Nonetheless, it remains a major challenge to construct multidrug regimens that are rapidly effective against drug-resistant TB, maintain more than one drug in the regimen at a locally effective concentration in all types of lesions over the dosing interval,^15^ are free of major drug–drug interactions, and are safe enough not to require monitoring for life-threatening toxicities.

The most widely used and impactful antibiotics in medicine are β-lactams, beginning with the historic introduction of penicillin into the clinic in the 1940s and continuing through the development of additional structural classes. In the decades following World War II, entire departments of some pharmaceutical companies were dedicated to the discovery and development of β-lactam drugs. In many companies, thousands of these compounds were produced, despite synthetic challenges, and tested against a wide range of Gram-positive and later Gram-negative bacteria. Efforts in these areas were focused initially on the penicillin (or penam) class, followed later by the cephalosporins (cephems), and finally the carbapenems, with each class offering new advantages primarily in potency and broad-spectrum activity.

β-Lactams are also among the safest antibiotics. β-Lactams disrupt the synthesis and promote the breakdown of peptidoglycan,^16^ the mesh-like macromolecule of covalently linked *N*-acetylglucosamine, *N*-acetylmuramic acid, and peptide chains of 3–5 amino acids (d-alanine, l-alanine, d-glutamine, l-lysine, and/or meso-diaminopimelic acid) that provides strength and shape to the bacterial cell wall. The restriction of peptidoglycan to bacteria contributes to the low toxicity of most β-lactams for eukaryotes.

After Fleming and others found penicillin to be inactive on Mtb,^17,18^ decades followed in which it was assumed that all members of the growing family of β-lactams would likewise be ineffective against Mtb. Mtb’s intrinsic resistance to β-lactams was attributed to its waxy outer cell wall and robust β-lactamase activity. In 1983, Cynamon and Palmer reported that β-lactamase inhibitors such as clavulanate could sensitize Mtb to β-lactam antibiotics *in vitro*.^19^ However, the conventional wisdom against the utility of β-lactams in the treatment of TB remained largely unshaken until early observations demonstrating that clavulanate potentiated beta-lactam efficacy were extensively confirmed and extended beginning in 2009.^19−28^ This led to the clinical demonstration in 2016 that coadministration of meropenem and clavulanate (the latter delivered in combination with amoxicillin) markedly reduced the burden of Mtb in the sputum of subjects over a two-week period.^20^

Although the early bactericidal activity of the combination of meropenem, amoxicillin, and clavulanate in patients with TB^20^ represents an advance for the field, meropenem’s potential for impacting TB treatment is limited by the requirements for intravenous administration and codosing with a β-lactamase inhibitor. Therefore, we decided to undertake a broad survey of β-lactams for their activity against Mtb in vitro. Since the discovery of penicillin, most major pharmaceutical companies have invested in β-lactam drug discovery. The outcome of this extensive effort can be observed within chemical databases in which tens of thousands of β-lactam analogues are described, the vast majority of which would never have been tested against Mtb due to the lack of testing capacity at the companies and the assumption of inactivity. We reasoned that a screen of this untapped chemical diversity could identify a suitable β-lactam scaffold for TB based on a number of factors. First, while the canonical β-lactam targets are penicillin-binding d,d-transpeptidases that catalyze 4 → 3 transpeptide linkages in peptidoglycan, Mtb also incorporates unusual peptidoglycan 3 → 3 linkages^29,30^ catalyzed by l,d-transpeptidases.^22,31−34^d,d- and l,d-transpeptidases differ in active site residues (serine vs cysteine, respectively) and therefore have different levels of susceptibility to different classes of β-lactams.^35−38^ Furthermore, a survey of β-lactams against Mtb should test the belief that β-lactams can only be highly active against Mtb when the pathogen’s major β-lactamase, BlaC, is inhibited by an agent such as clavulanate. Finally, this approach would also explore whether any β-lactams with improved pharmacokinetic profiles (increased half-life and/or oral bioavailability) could be identified for use against TB.

Additionally, such a survey could follow up on the surprising observation that some cephalosporins can kill Mtb selectively when it is nonreplicating,^39^ a state associated with phenotypic resistance to most antibacterials, including β-lactams. The essential role of peptidoglycan remodeling in cell growth and septation makes peptidoglycan synthesis a critical point of vulnerability during bacterial replication.^40^ However, some host microenvironmental conditions faced by Mtb—including acidic pH, hypoxia, oxidative and nitrosative stress, metal deprivation or intoxication, and lipid carbon sources—reduce or prevent replication.^41^ Of particular significance for reducing TB patients’ infectivity, when tested ex vivo, Mtb did not replicate in the caseum from lung cavities.^42^ Residence in the caseum and exposure to other conditions that impair Mtb’s growth are associated with partial or complete phenotypic resistance to most antibiotics.^41−46^ Thus, an additional motive for launching the project described below arose when β-lactams were identified that are cidal for both replicating (R) and nonreplicating (NR) Mtb in collaboration with the Tres Cantos Open Lab Foundation at the campus of GlaxoSmithKline (GSK) in Tres Cantos, Spain.^47^

Accordingly, we organized a project under the auspices of the TBDA to screen ∼8900 β-lactams from four major pharmaceutical companies—GSK, Sanofi, Lilly, and MSD. Screening was conducted by a scientist employed by Weill Cornell Medicine (WCM) working in the Open Lab at GSK and by other WCM scientists working at WCM; at the not-for-profit Infectious Disease Research Institute (IDRI); and at the National Institute of Allergy and Infectious Diseases (NIAID). Active compounds from the four screening sites were assembled at WCM and tested head-to-head. Chemical synthesis was carried out at the University of North Carolina (UNC), and pharmacologic studies were carried out at Rutgers University. Finally, the most promising compounds from the four companies were selected for in vivo studies at GSK. The pharma partners shared the structures of hundreds of their proprietary compounds with each other and with the nonpharma participants and jointly decided which compounds to prioritize for more complex studies.

Here, we describe strategies used to prioritize thousands of β-lactams from multiple companies into a small test set for further characterization. In contrast to the long-standing view that β-lactams are generally inactive against Mtb, we found that 18.0% were active at 50 μM with ≥90% inhibition against replicating Mtb in vitro. Moreover, analysis of a prioritized set of 307 β-lactams with replicating MIC_90_’s < 20 μM found that the activity of 11.7% was independent of the addition of a β-lactamase inhibitor. We identified numerous β-lactams with in vitro activity against Mtb and one that demonstrated in vivo efficacy in a dehydropeptidase-1 knockout model of murine TB. These hits represent attractive starting points for hit-to-lead efforts in future studies. Our work highlights both the advantages and challenges of the multipharma and multiacademic screening approach. This consortium model can provide a blueprint for others tackling infectious diseases.

## Results

### Primary Screening

**Figure 1:**
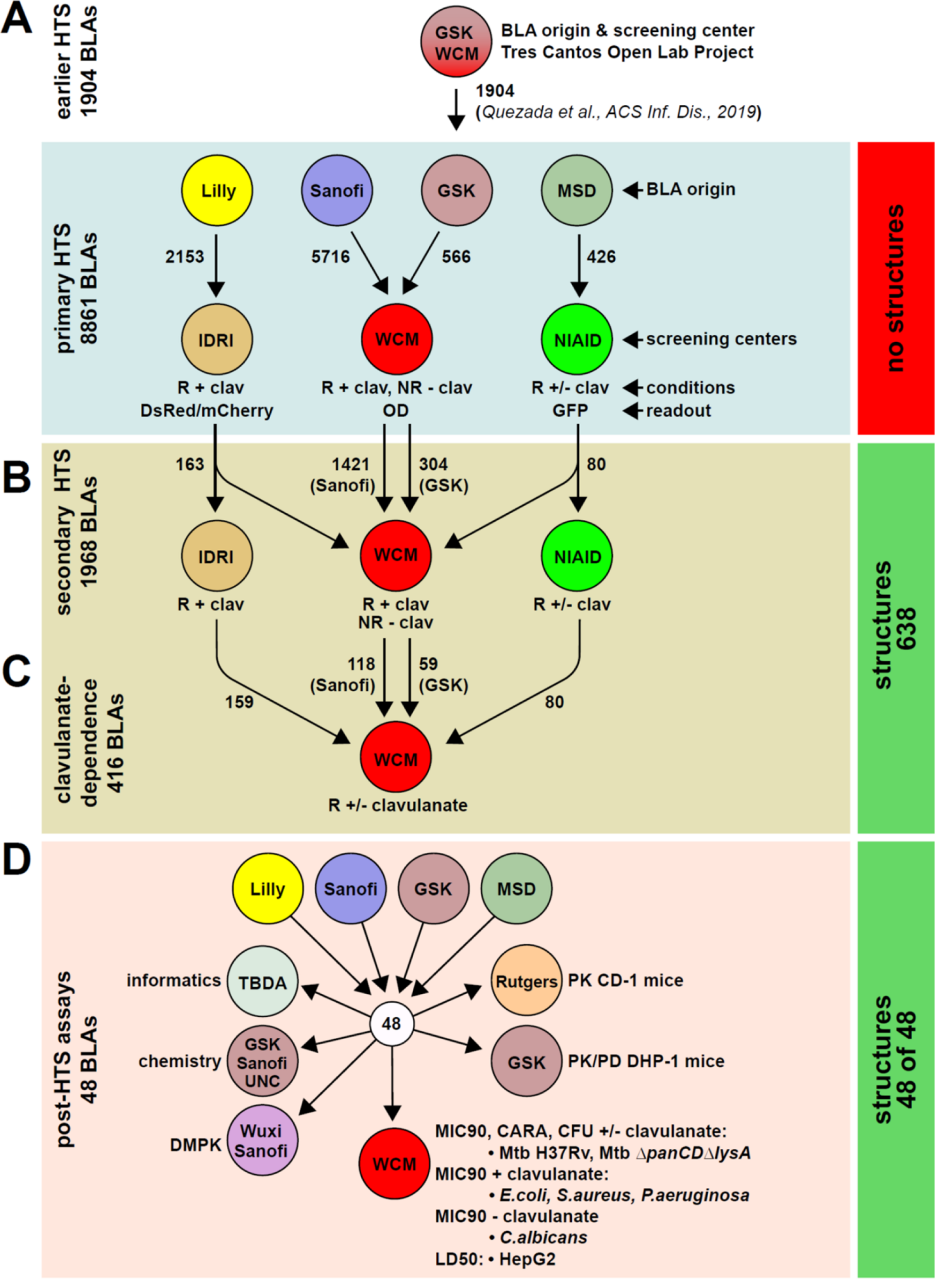
Schematic of the β-lactam screening strategy. (a) β-lactams were sent from four pharma partners to three academic screening centers to test for activity against wild-type H37Rv or Δ*panCD*Δ*lysA* Mtb that was replicating or rendered nonreplicating in a four-stress model.^48,50^ Primary screening hit rates are shown in Table S1. (b) Subset of actives from Sanofi and GSK were resupplied as fresh stocks to a single academic screening center (WCM) for retesting head-to-head against Mtb Δ*panCD*Δ*lysA* at WCM. The GSK primary and secondary screens included some β-lactams from an earlier screen of their β-lactam collection.^47^ Actives from Lilly and MSD were retested at the IDRI and NIAID, respectively. (c) 416 confirmed actives from all four companies were tested at WCM head-to-head for clavulanate dependency in the dose–response format against replicating Mtb Δ*panCD*Δ*lysA*. (d) Final set of 48 β-lactams supplied for PK, DMPK, chemistry, and phenotypic testing. The annotation on the right of the figure describes the structure reveal: in (a), no structures were disclosed; in (b,c), 638 structures were revealed to all parties; and in (d), the final 48 compounds were all assigned a structure (primarily those that were not assigned in (b,c). Abbreviations: GSK, Diseases of the Developing World at GlaxoSmithKline; IDRI, Infectious Disease Research Institute; WCM, Weill Cornell Medicine; NIAID, National Institute of Allergy and Infectious Diseases; UNC, University of North Carolina; TBDA, Bill & Melinda Gates TB Drug Accelerator; R, replicating conditions; NR, 4-stress nonreplicating conditions; clav, clavulanate at 4 μg/mL; DsRed/mCherry, red fluorescent protein; GFP, green fluorescent protein; and OD, optical density at A_580_.

We assembled ∼8900 β-lactams from GSK, Sanofi, Lilly, and MSD (Figure 1). Structures were blinded to all parties except the owners. This compound set represented essentially the entire diversity of β-lactams still available in these companies’ compound banks. All parties applied similar, yet independent, screening strategies to identify active β-lactams in a manner that leveraged their knowledge and expertise (Figure 1). At later stages, we agreed to pool compounds and collaborate closely during hit progression. All sites tested compounds in 384-well plates. Screening at GSK, the IDRI, and the NIAID was conducted under standard replicating (R) conditions against wild-type (WT) Mtb H37Rv. Primary screening at WCM was performed in duplicate against a pantothenate- and lysine-auxotroph of H37Rv (Mtb Δ*panCD*Δ*lysA*) under both R and nonreplicating (NR) conditions, low pH, hypoxia, nitrosative stress, and a fatty acid carbon source.^41,43,48−50^ To block the activity of Mtb’s primary β-lactamase, BlaC,^21^ all screening centers included clavulanate at 4 μg/mL for Mtb under R conditions.

WCM conducted the primary screening of 566 β-lactams from GSK and 5716 β-lactams from Sanofi; NIAID screened 426 β-lactams from MSD; and IDRI screened 2153 β-lactams from Lilly (Figure 1). Table S1 summarizes the results for the primary HTS campaigns. Using a ≥90% inhibition hit rate, the hit rates at the three screening centers under R conditions were 15.7, 30.1, 24.0, and 3.3% for Sanofi, GSK, Lilly, and MSD, respectively, averaging 18.0% (weighted by the total number of compounds screened).

### Hit Confirmation and Secondary Assays

All partners participated in selecting 1968 β-lactams for resupply and MIC_90_ (minimal inhibitory concentration leading to 90% inhibition of growth) determination. WCM determined MIC_90_ values for 1725 β-lactams from Sanofi and GSK under R conditions with 4 μg/mL clavulanate and under NR conditions without clavulanate (Figure 1b). A subset of the GSK β-lactams chosen for retesting was derived from an earlier β-lactam HTS run at the Tres Cantos Open Lab^47^ (Figure 1a). Similar dose–response testing of primary screening actives from Lilly and MSD was performed at the IDRI (163 compounds) and NIAID (80 compounds) under R conditions with 4 μg/mL clavulanate. Results are summarized in Table S2. When retested at WCM, 10.9% of β-lactams were inactive under either R or NR conditions (MIC_90_ > 100 μM and <50% inhibition of growth at 100 μM). Testing under both R and NR conditions at WCM (Figure S1) permitted a tentative assignment of the activity spectrum for β-lactams from Sanofi and GSK. We identified 492 selectively R-active and 36 candidate dual-active β-lactams (92.0 and 6.7% of the active molecules, respectively).

**Figure 2:**
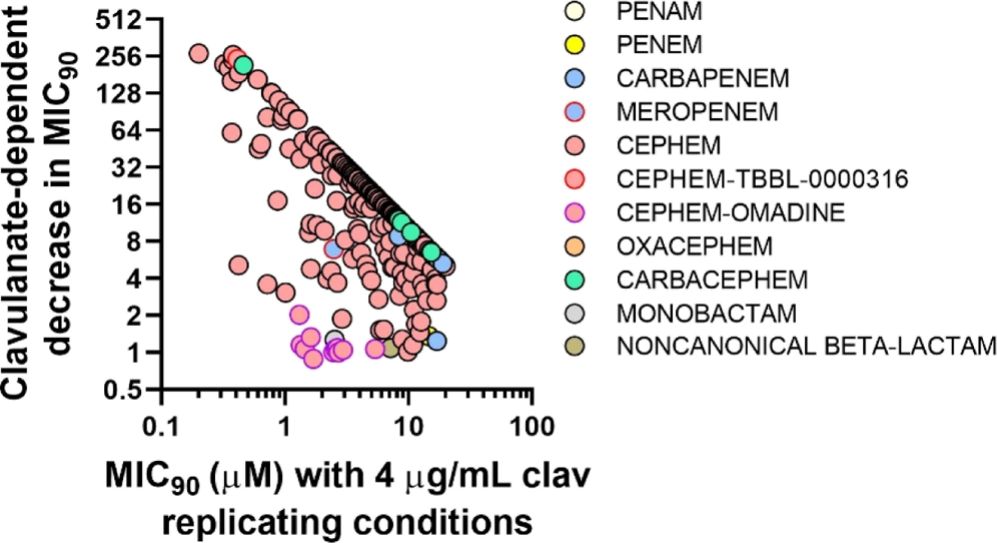
Clavulanate dependency of Mtb-active β-lactams. β-Lactams with an MIC_90_ of ≤ 20 μM in the presence of 4 μg/mL clavulanate against replicating Mtb were evaluated for their MIC_90_ shift in the presence and absence of clavulanate (Figure 1c). The fold shift calculation was limited by the maximum concentration tested (100 μM). Meropenem was used as a control molecule that reproducibly showed a clavulanate shift of ∼8-fold.^21^

Recognizing that each screening site/assay was expected to produce slightly different results, the partners agreed on the essential step of centralizing secondary screening at a single site. WCM was chosen for this task. At this stage, 418 of the R-active β-lactams were resupplied from the four pharma teams as fresh stocks to test clavulanate dependency and to compare the compounds head-to-head on the same day at WCM (Figures 1c; 2; Table S3). Of these 418 compounds, 11.7% had a ≤4-fold increase in potency against replicating Mtb in the presence of clavulanate.

### Sharing of Compound Structures

**Figure 3:**
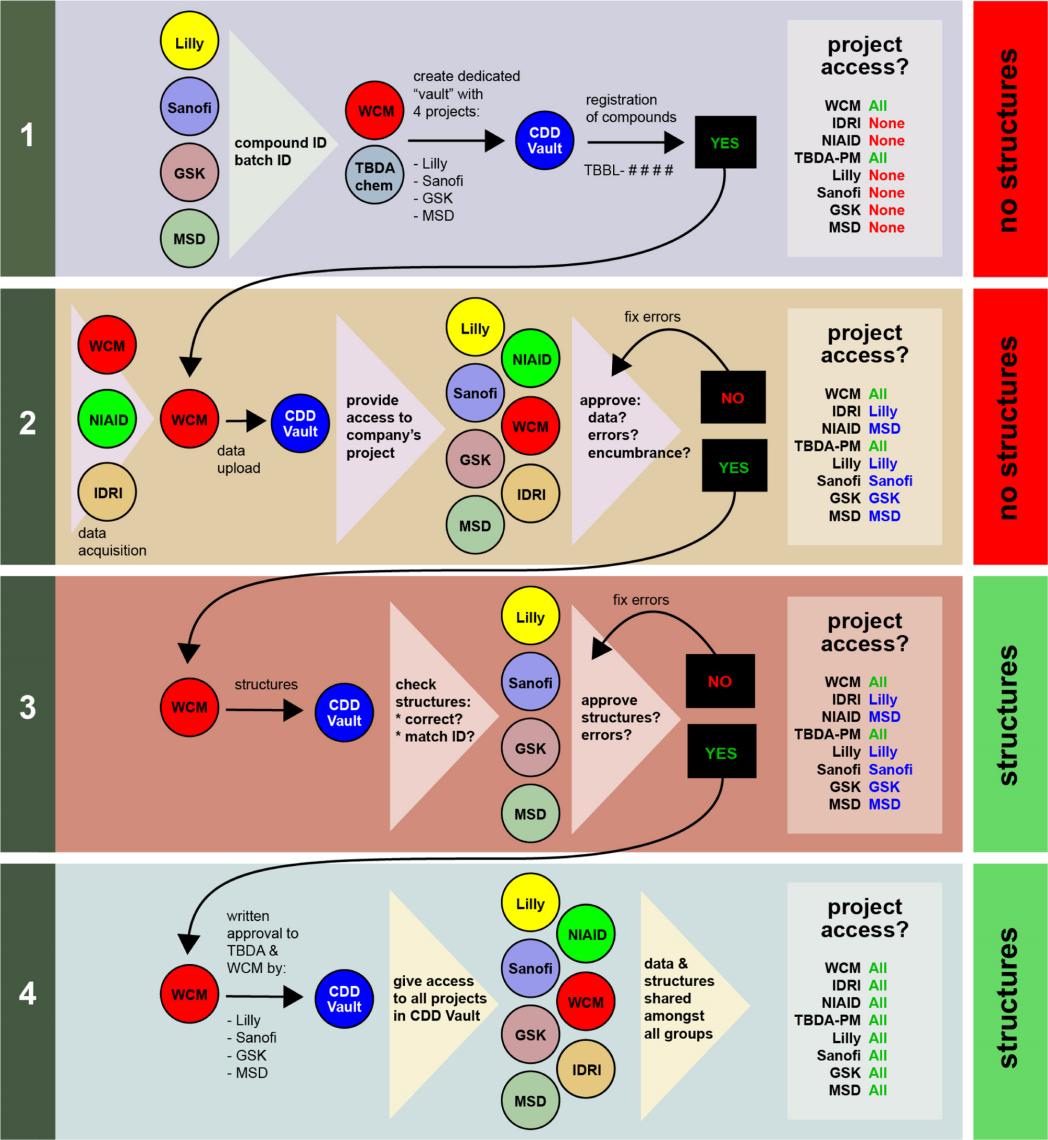
Overview of the pharma–academic consortium model. Access to structures is tiered using a four-step process: (1) Structureless compounds are registered into a private vault within CDD; (2) Registered, structureless compounds in CDD Vault are then associated with data; (3) Compound IDs and batches are then associated with structures; (4) pharma partners grant permission to share structures and data with all consortium partners, including the other academic groups (NIAID and IDRI). Access to the CDD Vault projects is carefully tiered in steps 1–4 and ultimately results in complete access to structures and data by all consortium members. Key: TBDA-PM = TB Drug Accelerator program manager.

While the use of blinded structures was required for working with large numbers of compounds from the different companies, the partners recognized early on that structure sharing would be necessary to facilitate compound progression and to ensure that the screening hits did not contain duplicates or close structural analogues. Therefore, we designed a mechanism to disclose a subset of structures representing the 1968 primary screening actives at an early stage via a four-stage system of access (Figure 3).

We created a TBDA β-lactam data vault at Collaborative Drug Discovery (CDD) (Figure S2) that was overseen by the medicinal chemist who serves as the TBDA’s data coordinator. In stage 1 (Figure 3, step 1), pharma partners provided structureless compound identifiers (including a unique batch ID) that were registered in CDD Vault and given a unique TBDA beta-lactam (TBBL) number. CDD Vault recorded each compound’s ownership, ID, and batch ID as metadata. Within the TBDA β-lactam vault, we created a private project folder for each pharma partner (Figure S2). At this stage, only WCM, the TBDA coordinator, and a CDD Vault consultant could access the four project folders.

In stage 2 (Figure 3, step 2), dose–response data and the derived MIC_90_s generated at WCM were uploaded into the project folders at CDD Vault. After WCM verified or corrected the automated curve fitting, each pharma partner was granted access to its own project folder to check for errors (e.g., to confirm that the batch IDs corresponded to the compound IDs). At this point, each company also reviewed each compound in its project folder for encumbrances. In stage 3 (Figure 3, step 3), companies sent structure information in the SMILES format in a password-protected Microsoft Excel file to WCM, the TBDA data coordinator, and the CDD representative. When the compound files in CDD Vault were updated with structural information by matching compound and batch IDs, pharma partners reviewed their project folders to confirm or correct features such as the structure, stereochemistry, and salt form and to double-check their authorization to share the structures.

In Stage 4 (Figure 3, step 4), the pharma partners affirmed in writing that they had reviewed the structures and associated data of their compounds for accuracy. Of the 1968 compounds slated for the dose–response studies, they agreed to allow all the collaborators to view 638 structures and associated data. Criteria used in determining which structures to share included replicating activity, purity, and lack of encumbrances.

### Structural Comparison of β-Lactams

Although we anticipated a high degree of structural redundancy in Mtb-inhibiting β-lactams from the four pharma collections, we were pleased to find almost no compound duplicates (Figure S3a) and little structural similarity (Figure S3b) among the compounds from the four companies.

GSK’s β-lactams were 95% cephalosporins. This heavy bias was driven by overall compound availability and purity being much higher for this class than for others. While the penam class was more represented within the overall GSK collection than cephalosporins, most of the penems were older and not available for testing. The remaining 5% of compounds supplied by GSK were evenly distributed among penems, carbapenems, monobactams, and a small number of β-lactam surrogates. Sanofi’s β-lactams were 59% cephalosporins, 15% penicillins, 1.3% monobactams, < 0.1% carbapenems and penems, and 24% other β-lactam classes. Lilly’s β-lactams were ∼80% cephalosporins and 10% penicillins, while the remaining 10% consisted of a mixture of other β-lactams. MSD’s β-lactams were 66% cephalosporins, 9% carbapenems, and 25% penicillins. Of the all the β-lactam structural classes revealed by the pharma partners for comparison (8532 total), there were approximately 67% cephalosporins, 13% penicillins, 0.8% monobactams, 0.5% penems and carbapenems, and 18% other β-lactam classes. While the compounds’ diverse chemical properties covered a large amount of chemical space (Figure S4), the final assembled library consisted predominantly of cephalosporins. The predominance of cephalosporins in the screening set likely reflects their prevalence in β-lactam discovery efforts during the 1970’s and 1980’s, along with their higher chemical stability relative to that of other β-lactam classes.

### Characterization of β-Lactams

After taking into account the compounds’ activity profile, availability, purity, and stability, the collaborating medicinal chemists nominated 48 R-active β-lactams, of which 9 were both R- and NR-active, for secondary assays (Figure 1d; Table S4). WCM scientists then determined the compounds’ MIC_90_’s against both Mtb Δ*panCD*Δ*lysA* and wild-type Mtb H37Rv under R conditions and under the 4-stress NR conditions, in both cases with and without 4 μg/mL clavulanate. WCM scientists then used the charcoal agar resazurin assay (CARA) to provide a semiquantitative estimation of the concentration of compounds leading to ≥99.0–99.9% reduction in colony-forming units (CFU) of WT Mtb H37Rv.^49,51^ Most of the β-lactams were predicted to be bacteriostatic by the criterion of causing ≤3 log_10_ kill by day 7.^51^ In addition, they had a narrow antimicrobial spectrum (Figures S5 and S6, and Table S4), and the majority were nontoxic to the human hepatoma cell line HepG2 (LD_50_ ≥ 100 μM) (Table S4).

### Dual-Active β-Lactams

**Figure 4:**
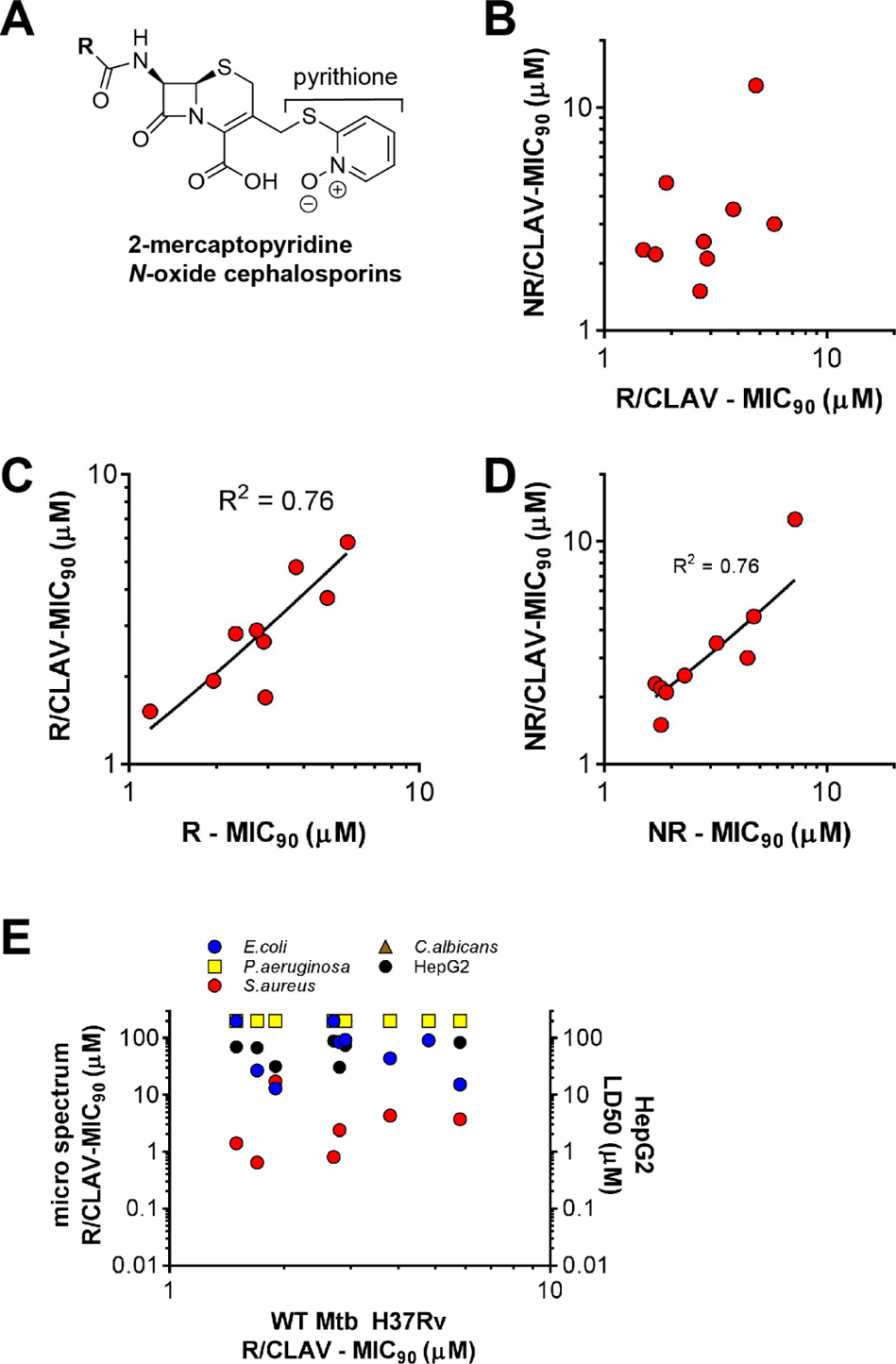
Dual-active β-lactams. Numerous pyrithione (2-mercaptopyridine *N*-oxide) cephalosporins (a) display similar activity against (b) replicating and nonreplicating wild-type Mtb H37Rv and in the presence or absence of 4 μg/mL clavulanate under (c) replicating or (d) nonreplicating conditions. (e) Microbial spectrum. The majority of pyridine-cephalosporins were active against *Staphylococcus aureus* but not other bacteria or fungi and had minimal toxicity to human HepG2 cells. Values > 100 μM in the microbial spectrum and toxicity assays in (b–e) were graphed as 200 μM, and in some cases, data points were obscured by other data points.

We defined dual activity as activity against both R and NR Mtb^41^ when the NR activity was confirmed using CARA and/or CFU assays.^51^ We recently identified three dual-active β-lactams from the GSK β-lactam collection that were cephalosporins bearing a 2-mercaptopyridine *N*-oxide moiety (also called omadine or pyrithione) at the C3′ nucleofugic position (Figure 4a).^47^ These compounds were included in our current screening and follow-up studies. Six more dual-active β-lactams were identified in the screening and their structures were shared. As recently reported, both the pyrithione, when released upon cleavage of the β-lactam ring by β-lactamases or by target engagement, and the residual portion of such molecules can contribute to antimicrobial activity.^47^

Most of these compounds had MIC_90_’s < 10 μM that were similar under either R or NR conditions (Figure 4b), and their activity was clavulanate-independent under both R (Figure 4c) and NR (Figure 4d) conditions. Additionally, 67% (6 of 9) had sub-10 μM MIC_90_’s against *S. aureus* and weak or no activity against *Escherichia coli*, *Pseudomonas aeruginosa*, and *Candida albicans* (Figure 4e). The pyrithione-containing cephalosporins were nontoxic to HepG2 cells (Table S4). Of the other 37 R-active β-lactams whose NR activity was evaluated by the CARA, none possessed dual activity. Thus, the ability to kill both R and NR Mtb (in a 4-stress model) was limited to cephalosporins bearing a pyrithione moiety.

### R-Active β-Lactams

**Figure 5:**
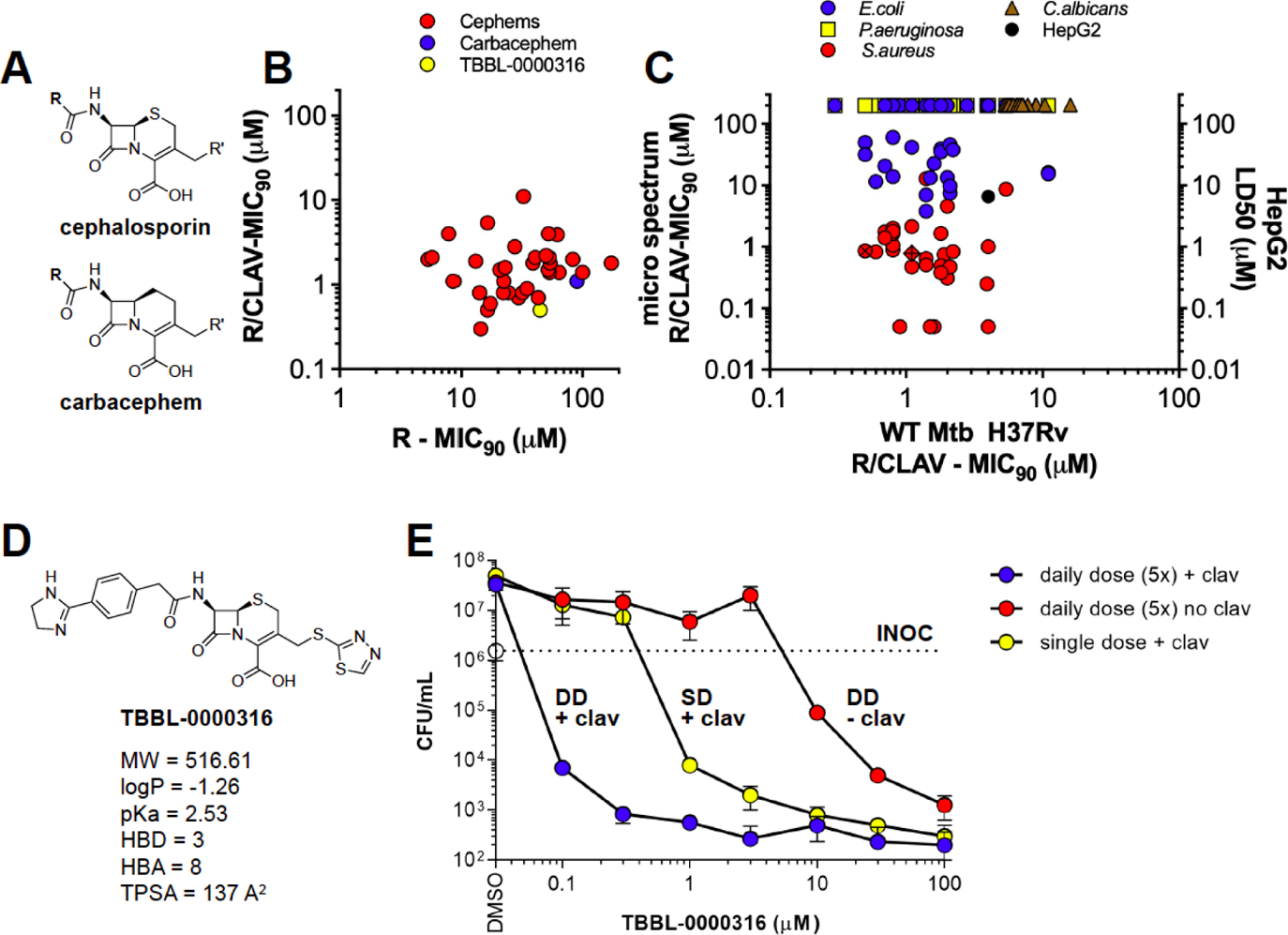
Replicating-active β-lactams. Generic structures of (a) cephalosporins and carbacephems. (b,c) Activity of compounds determined against replicating Mtb H37Rv in the presence and absence of clavulanate. (c) Activity tested against a panel of microbes in the presence of 4 μg/mL clavulanate. Toxicity was determined against HepG2 cells. (b,c) Data shown for R-active compounds with MIC_90_ < 20 μM. In (c), the hatched red circle is TBBL-0000316 and the hatched red diamond is the carbacephem. Structure of (d) TBBL-0000316. (e) When provided as a single dose, the combination of TBBL-0000316 and 4 μg/mL clavulanate killed replicating WT Mtb H37Rv. TBBL-0000316 potently killed replicating, virulent Mtb H37Rv in a clavulanate-dependent manner when dosed daily for five days. In (e), cells were plated on day 0 (inoculum = INOC) or day 7 and CFU was enumerated on 7H11-OADC bacteriologic agar plates for 3–4 weeks.

Of the final set of 46 compounds active against R Mtb (with an MIC_90_ of < 20 μM in the presence of clavulanate, including the dual-active beta-lactams), 45 (97.8%) were cephalosporins and 1 (2.2%) was a carbacephem (Figure 5a). Most compounds selectively active against R-Mtb had ≥ 4-fold MIC_90_ shift with the addition of clavulanate (Figure 5b). Over 20 of them had activity that was equipotent to or greater than that of meropenem. A total of 38% of the β-lactams had potent activity (MIC_90_ < 1 μM) against *S. aureus*, 14% had modest activity (MIC_90_ < 10 μM) against *E. coli* (Figure 5c), and none was active against *Salmonella enterica*, *P. aeruginosa*, or *C. albicans*. All but one of the R-active cephalosporins were nontoxic to HepG2 cells (Figure 5c).

### R-Active Cephalosporin TBBL-0000316

Given its potent activity against Mtb, lack of toxicity, and encouraging DMPK results detailed below, the cephalosporin amidine TBBL-0000316 (Figure 5d) was chosen for additional profiling using a CFU assay. In the presence of clavulanate, TBBL-0000316 had potent activity against replicating Mtb Δ*panCD*Δ*lysA* (MIC_90_ = 0.2 μM) and virulent Mtb H37Rv (MIC_90_ = 0.5 μM). In the absence of clavulanate, TBBL-0000316 had weak activity against wild-type Mtb and Mtb Δ*panCD*Δ*lysA* (MIC_90_ of 44.7 μM and 52 μM, respectively). TBBL-0000316 was nontoxic to human HepG2 cells (IC_50_ > 100 μM) and had weak to no antimicrobial activity against *P. aeruginosa*, *E. coli*, or *C. albicans* (MIC_90_ > 100 and 31.8 and >100 μM, respectively) but showed sub-micromolar activity against *S. aureus* (MIC_90_ = 0.86 μM with 4 μg/mL clavulanate). After a one-week exposure in the presence of 4 μg/mL clavulanate, a single dose of TBBL-0000316 had a bacteriostatic effect between 0.3 and 1 μM, while at 10 μM, it led to a 3.5 log_10_ reduction in CFU (Figure 5e). Due to the intrinsic lability of the β-lactam ring, we determined the impact of adding fresh TBBL-0000316 daily for five sequential days. Daily dosing of TBBL-0000316 and 4 μg/mL of clavulanate for 5 days led to a 3–4 log_10_ reduction in CFU at ≥1 μM (Figure 5e).

To identify the beta-lactams with properties predictive of suitability for animal studies, we compared the pharmacologic features of TBBL-0000316 and 22 other β-lactams from our screen (Table S5). Beta-lactams were nominated based on their activity relative to that of meropenem, activity profile (R-active vs dual-active), structural diversity, and stock availability and selected by consensus of the consortium members. Meropenem and ertapenem served as controls. Because increased human plasma protein binding and plasma stability are characteristics of longer acting β-lactams and chemical stability and membrane permeability are associated with improved oral bioavailability,^52^ we tested for stability in fasted-state biorelevant media simulating small intestinal fluid (FaSSIF), simulated gastric fluid (SGF), and mouse, rat, dog, monkey, and human plasma; binding to mouse, rat, dog, monkey, and human plasma proteins; and Caco-2 permeability. Moderate plasma stability at 120 min was considered a critical parameter for predicting in vivo activity. Only a few compounds gave Caco-2 permeability results predictive of oral absorption; however, testing oral PK was still favored in the event that one or more β-lactams gave sufficient exposure. The final selection of compounds for in vivo PK was made based on the minimal criteria of stability in plasma and then by attempting to balance potency under different conditions with improved PK profiles.

In these in vitro studies, TBBL-0000316 demonstrated desirable properties for efficacy studies: 2-h plasma stability: 89, 86, and 86% (% remaining; mouse, rat, and human); plasma protein binding: 18, 62, and 38% (mouse, rat, and human); and FaSSIF stability: 95 and 84% remaining after 6 or 24 h, respectively. However, TBBL-0000316 was anticipated to have poor oral bioavailability (Caco-2 permeability = 3 nm/s). For reasons described below, TBBL-0000316 was subjected to further characterization, including solubility at pH 1 (402 μg/mL) and pH 7.4 (>1000 μg/mL), hERG inhibition (IC_50_ > 50 μM), and metabolic stability (μL/min/mg protein (1/2 life, minutes): human = 5.4 (256) and mouse = 1.1 (1300)). TBBL-0000316 neither showed inhibition of CYP3A4 in the presence or absence of NADPH (IC_50_ > 25 μM) nor did it induce expression of CYP1A2, CYP2B6, or CYP3A5 (all <2-fold at 3 μM). These DMPK results led to the nomination of TBBL-0000316 and six additional β-lactams for testing in CD-1 female mice by both intravenous (IV, 5 mg/kg) and oral (PO, 25 mg/kg) dosing. Dosed orally to CD-1 mice, all 7 β-lactams had short half-lives, poor exposure (97–1356 h × ng/mL), and low bioavailability (0.11–1.33%). Dosed intravenously, many had moderate half-lives (0.38–0.78 h) and exposure (3330–39113 h × ng/mL) (Figure S7 and Table S6).

**Figure 6:**
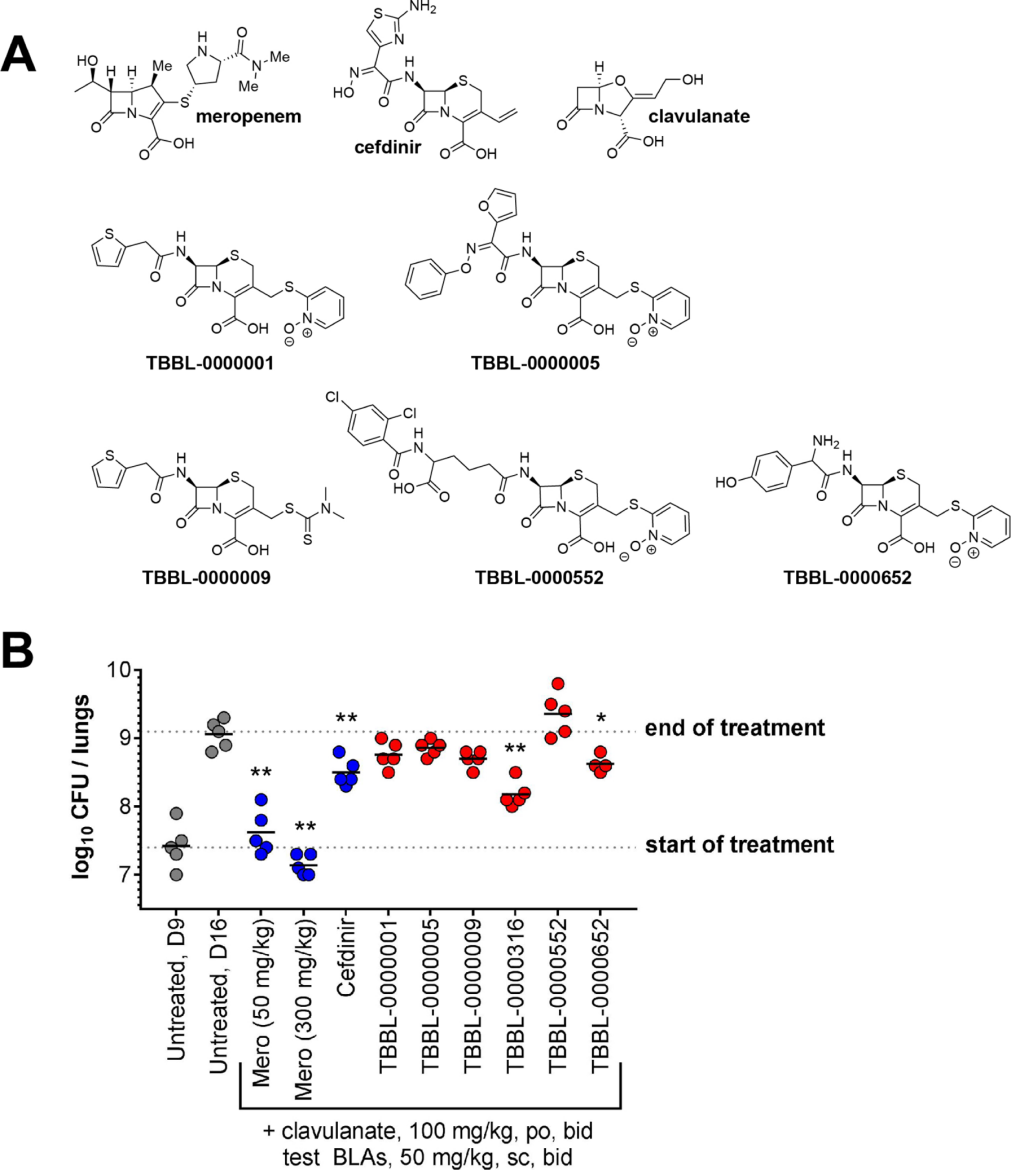
Activity in a DHP-1 model of murine TB. (a) Structures of β-lactams tested in a dehydropeptidase-1 (DHP-1) knockout mouse model of acute TB. The structure of TBBL-0000316 is in Figure 5d. (b) Compounds were provided to infected mice between days 9 and 15 of the infection and CFU was enumerated by plating lung homogenates on 7H11-OADC agar plates supplemented with 0.4% activated charcoal to prevent drug carryover. Unless otherwise noted, β-lactams were dosed BID at 50 mg/kg SC and paired with clavulanate-dosed BID at 100 mg/kg PO. *P*-values were obtained by ANOVA analysis (Dunnett’s post-test, GraphPad software) comparing treatments with test agents with the day 16 CFU counts from untreated mice. The fates of individual mice are summarized in Table S9. *p < 0.01–0.05, **p = 0.001–0.01.

Mice hydrolyze some β-lactams more rapidly than humans.^53,54^ To improve exposure, we used dehydropeptidase-1 (DHP-1)-deficient mice^54,55^ given 100 mg/kg clavulanate (po). When dosed subcutaneously (sc) at 300 mg/kg into uninfected DHP-1 knockout mice, the β-lactams (Figure 6a) achieved a high *C*_max_ (2647–29,900 ng/mL) and exposure (8811–61,949 h × ng/mL) (Figure S8; Table S7). Since we observed nonlethal toxicity in the first study with uninfected mice, we used a lower dose level (50 mg/kg sc) in a second study to test the efficacy in Mtb-infected DHP1 knockout mice (Figure 6, Tables S8, S9). Meropenem and cefdinir were used as positive controls. Meropenem had a bacteriostatic impact (*p* > 0.05, ANOVA, Dunnett’s post-test, compared to day 9, the start of treatment), while cefdinir showed a lower lung CFU count compared to untreated mice at day 16 (end of treatment), as reported (Figure 6b).^55^ Two of the test β-lactams inhibited Mtb growth (Figure 6b) with statistical significance. The cephalosporin amidine TBBL-0000316 reduced CFU by 0.9 log_10_ (*p* < 0.001) compared to untreated control mice, and the dual-active *N*-oxide cephalosporin TBBL-0000652 reduced CFU by ∼0.5 log_10_ (*p* < 0.05). TBBL-0000009 reduced CFU by ∼0.4 log_10_ (close to statistical significance, *p* = 0.052).

## Discussion

This collaboration demonstrated that multiple major pharmas, academics, not-for-profits, and government institutions can work noncompetitively and productively on a major problem in anti-infectious drug discovery. Collaboration was facilitated by the mediation of a foundation and the use of an independent data-sharing facility. Medicines for Malaria Venture operates comparably.^56^ To the best of our knowledge, however, this is the first post-World War II effort in antibacterial drug discovery that has involved joint inspection by multiple pharmas of each other’s proprietary compounds and collective prioritization of compounds for progression. Ironically, the compound class involved is the same as that in the Penicillin Project of World War II.^5^

A consortium approach to drug discovery that includes head-to-head comparison of multiple corporate compound collections at a single site may allow access to greatly increased chemical diversity. Although this poses the theoretical risk of wasting resources by studying compound sets that are substantially redundant, the Mtb-active β-lactams from four major pharmas whose structures were shared included only one compound owned by all four of them. High structural similarity is typically determined with a Tanimoto correlation coefficient of 0.7; however, due to the intrinsic nature of β-lactam structural similarity resulting from a shared pharmacophore, we looked for high structural similarity by applying a Tanimoto coefficient of >0.85. Using this strict criterion, there were no highly similar β-lactams across all four collections. Structurally similar β-lactams were shared by no more than two of the four firms, and the highest proportion shared by two firms was 12.6%. This experience endorses the premise that multipharma collaboration can afford access to increased chemical diversity.

Multiparty drug development consortia present challenges related to the need to guard proprietary structures of compounds before review of their potential encumbrances, such as relevance to other internal drug development campaigns or contractual obligations to other parties. We surmounted these challenges with a system of tiered access characterized by multiple checkpoints for quality control (QC) and authorization. Our workflow leveraged academic–pharma collaborations already existing in the TBDA (GSK/WCM; Sanofi/WCM; Lilly/IDRI; and MSD/NIAID) to facilitate rapid compound distribution and immediate initiation of the primary HTS. As we identified smaller numbers of prioritized compounds, each pharma partner shared compounds for testing head-to-head at a single facility.

Early compound selection and decision making were driven by the individual pharma partners together with their academic screening center. Selections were based primarily on compound quality, availability, and Mtb potency, although encumbrances were also checked. Once screening was centralized at WCM and compound structures had been shared, decision making became consensus-based. No significant conflicts arose via this method as scientific data led to clear decision points in most cases. For a handful of compounds at the borderline of certain criteria, the original pharma provider made the call whether to proceed. Although having many collaborators take part in decision making risked slowing progress, it helped maintain partner engagement. For progression of these compounds in a hit-to-lead campaign and beyond, a smaller team would be appropriate.

Historically, the poor activity of β-lactams against Mtb has been attributed to poor permeability, an intrinsic resistance of Mtb transpeptidases to inhibition by β-lactams, and cleavage by β-lactamase; however, recent data and this study indicate that a major determinant of β-lactam antimycobacterial activity is whether or not a β-lactamase inhibitor was present.^20,21,28,47,57−61^ Reversing conventional understanding, the present effort revealed that hundreds of β-lactams are active against replicating Mtb *in vitro*—between ∼3 and 30% of those we tested, depending on the compound collection. The proportion stands in striking contrast to the usual hit rate for a high-throughput screen of pharma compound collections against Mtb, which rarely exceeds 0.1–1%. The proportion of Mtb-active β-lactams might have been higher if compound degradation had not been widespread. Moreover, the requirement for inhibition of β-lactamase was far from universal, with ∼12% of the R-active β-lactams we tested being largely indifferent to the addition of clavulanate. The majority of clavulanate-independent β-lactams were dual-active omadine-cephalosporins.^47^ The structural biases of β-lactam libraries constructed to identify broad-spectrum antibiotics against R bacteria might explain why we identified only a few β-lactams active against Mtb rendered NR by the multistress assay. The NR activity of some β-lactams may be attributable to the disruption of the function of noncanonical targets.^39,47,62,63^

Cephalosporins dominated the classes of β-lactams we identified as Mtb-active. To some extent, this likely reflects the relatively high stability of the cephalosporin class versus other β-lactams, particularly after decades of storage. However, cephalosporins might have physicochemical properties favoring entry into Mtb’s periplasm or affinity for the relevant targets, not all of which may be transpeptidases. We identified cephalosporins with selective activity against replicating Mtb, nonreplicating Mtb,^39^ or both replicating and nonreplicating Mtb.^47^

Although the mouse rapidly hydrolyzes β-lactams, we identified a novel, potent cephalosporin, TBBL-0000316, with activity against Mtb in the mouse, enriching the short list of β-lactams, mostly carbapenems, reported to have in vivo activity against Mtb: meropenem, faropenem, and cefdinir.^55^ Furthermore, this is the first report demonstrating the activity of omadine-cephalosporins^47^ in murine TB.

Our collaborative findings add to an increasing body of evidence that β-lactams are a rich source of potential antibiotics whose potency and lack of toxicity make them attractive for the treatment of TB.^20,21,47,55,64^ Further progression of the most advanced β-lactam studied here, the cephalosporin amidine TBBL-0000316, would require optimization to improve its DMPK profile,^52^ particularly its oral bioavailability and half-life. These optimization goals represent significant challenges, given the decades of previous efforts to enhance the properties of this critical drug class. However, none of this historic effort was directed against a disease for which oral agents are as critical as with TB, so it can be argued that the incentives for further research were not there. Additionally, the underlying enzymatic biology of how β-lactams interact with the transpeptidase targets of *Mtb* is only beginning to be unraveled. Combining this emerging biology with modern, computationally powered drug discovery approaches could offer new hypotheses for the optimization of TBBL-0000316 or other hits from this screening effort.

Most importantly, this study may serve as a blueprint for future academic–pharma and pharma–pharma collaborative efforts in anti-infective drug discovery.

## Methods

### Strains and Growth Conditions

Mtb H37Rv was grown in Middlebrook 7H9-ADN-Tyl (0.2% glycerol, 0.5% albumin, 0.2% dextrose, 0.085% NaCl, and 0.02% tyloxapol) or Middlebrook 7H9-OADC-Tyl or 7H9-OADC-Tw (0.2% glycerol (WCM; not added at IDRI), oleic acid, albumin, dextrose, catalase, 0.02% tyloxapol (WCM), or 0.05% Tween80 (IDRI)) with incubation at 37 °C with 5% CO_2_ and 20% O_2_. To use robotics in a BSL2+ facility, the attenuated strain, Mtb Δ*panCD*Δ*lysA*, was used for screening at WCM.^48^ Mtb Δ*panCD*Δ*lysA* was grown in Middlebrook 7H9-OADC-Tyl (0.2% glycerol, oleic acid, albumin, dextrose, catalase, and 0.02% tyloxapol) with the addition of 24 μg/mL pantothenate, 240 μg/mL l-lysine, and 0.5% CAS amino acids. The screening strain Mtb H37Rv::pMSP12 expressing a cytosolic green fluorescent protein^65^ was grown in 7H9 supplemented with 0.2% glycerol, 10% ADN supplement, and 0.05% Tween 80. The replicating and nonreplicating high-throughput screening methods were as described in the literature.^43,48−51^ Mtb was rendered NR in modified Sauton’s medium (per liter: 0.5 g of KH_2_PO_4_, 0.5 g of MgSO_4_, 0.05 g of ferric ammonium citrate, 0.5% BSA, 0.085% NaCl, and 0.02% tyloxapol) at pH 5.0 with 0.5 mM NaNO_2_ and 0.05% butyrate and incubated at 1% O_2_ and 5% CO_2_. For NR assays, Mtb in the mid-log phase was washed twice in Dulbecco’s PBS containing 0.02% tyloxapol and resuspended at an OD_580_ of 0.1 in NR medium. Replicating Mtb was used at an OD_580_ of 0.01. To evaluate the microbial spectrum of β-lactams,^48^ the following strains were grown at 150 μL in a TC-treated 96-well microplate in Luria broth: uropathogenic *E. coli* TOP10, *S. enterica* var. Typhimurium, and *P. aeruginosa* PA01; in Mueller–Hinton broth: *S. aureus* American Type Culture Collection (ATCC) 29213; and YM broth at pH 5.5: *C. albicans* ATCC 90028. Clavulanate (4 μg/mL) was included in the assays with the four species of bacteria.

### Assembling a β-Lactam Library

The majority of β-lactams from GSK, Sanofi, Lilly, and MSD were synthesized decades ago, and storage conditions varied widely. QC tests revealed that compound degradation was common. Thus, the first major hurdle that we faced was identifying β-lactams that passed a minimal QC threshold. Each pharma team used its own criteria for choosing which β-lactams to submit. Sanofi provided ∼55% of their total β-lactam corporate collection on hand as solids in amounts >2 mg. A small test set was used for QC, with the goal of >80% purity. GSK ran QC on 619 DMSO stocks of β-lactams; 58% exceeded 50% purity and were sent to WCM for screening. In addition, fresh solutions were prepared from 203 solids chosen to represent β-lactam diversity within the GSK collection. Only 20% of these samples passed QC and proceeded to screening. Lilly identified 2153 lactam-containing molecules with solid inventory based on searches of 9 substructures (cephalosporin, penicillin, and related). To preserve their stability, ∼500 selected molecules were freshly solubilized and shipped within two weeks to IDRI for screening. The most promising 163 compounds, based on the structure and activity, were then tested for QC by Lilly, using liquid stocks 4 months after dissolution. QC revealed that 30% of compounds were >90% pure and 50% were >80% pure. Of 1041 β-lactams identified in MSD’s collection, 411 (40%) were found to be >80% pure.

### Screening of β-Lactams

β-Lactams were stored as powders or DMSO stocks. Samples of a limited number of freshly prepared DMSO stocks or thawed DMSO stocks were assayed for purity by LC–MS. Compounds were assayed for activity against R and NR Mtb in the 384-well format as described previously for WCM,^48^ IDRI,^66,67^ NIAID,^68^ and GSK.^47^ Freshly prepared clavulanate was included at 4 μg/mL. At WCM, compounds were tested at 50 μM, recording inhibition of growth of Mtb by optical density after 7 days under R conditions or upon dilution under R conditions following 7 days of exposure to the compound under NR conditions. Based on pilot experiments, we omitted clavulanate from tests under NR conditions. β-Lactams at GSK were screened using similar methods.^47^ At NIAID, the primary screening was performed using replicates in a dose–response format (0.04−μM) in the presence and absence of clavulanate against an H37Rv strain overexpressing a cytosolic green fluorescent protein. The NIAID retested β-lactams in a 96-well format. At IDRI, the primary screening was performed at 50 μM against H37Rv expressing DsRed/mCherry. Mtb was exposed to compounds for seven days, and viability was estimated by OD_580_ or fluorescence (480 ex; 580 em).

Hit criteria chosen by each team were similar. Lilly–IDRI chose ≥90% inhibition under R conditions; MSD–NIAID chose an IC_50_ of <50 μM in a 7-day exposure period against Mtb:pMSP12 growing in 7H9/ADC/Tw in the presence or absence of 4 μg/mL clavulanate in a 12-point dose response ranging from 0.04 to 50 μM; and WCM used the following criteria: R active: ≥80% inhibition under R conditions and ≤50% inhibition under NR conditions; strict NR active: ≥80% inhibition under NR conditions and ≤50% inhibition under R conditions; and candidate dual active: ≥80% inhibition under both R and NR conditions.^49^

### Minimal Inhibitory Concentration Assays (MIC_90_)

Compounds were tested in the presence or absence of 4 μg/mL clavulanate in twofold serial dilutions (from 0.2 to 100 μM) against the following Mtb strains (screening center locations): (1) at WCM: H37Rv or Δ*panCD*Δ*lysA* (WCM) and measuring OD_580_ on day 7; (2) at IDRI: Mtb H37Rv (ATCC 25618) constitutively expressing *Ds*Red^67^ and measuring fluorescence on day 5;^66^ and (3) at NIAID: Mtb H37Rv by measuring GFP fluorescence from pMSP12 on day 7. NIAID additionally tested the top 82 β-lactams in a 96-well plate format against wild-type Mtb H37Rv. MIC_90_ was defined as the minimal inhibitory concentration of the test agent leading to 90% inhibition of growth at day 7.

### Determination of Cytotoxicity

Compounds were tested for cytotoxicity against HepG2 cells as previously described.^48,69,70^ Briefly, cells were exposed to compounds for 48–72 h, and viability was measured using CellTiter-Glo (Promega).

### Charcoal Agar Resazurin Assay (CARA)

Semiquantitative evaluation of bacterial viability using the CARA was performed as described previously.^49,51^ Briefly, R and NR MIC_90_ assay plates were mixed, and 10 μL of cultures was sampled and dispensed onto the surface of 96-well tissue culture-treated microplates containing 200 μL of 7H11-OADC-tyloxapol and 0.4% (w/v) activated charcoal (“CARA microplates”). CARA microplates were then incubated at 37 °C with 1% O_2_ and 5% CO_2_. After 7–10 days of incubation, plates were developed by the sequential addition of 40 μL of 7H9-OADC-glycerol-tyloxapol to premoisten the cells and agar and then 40 μL of developing agent (0.01% resazurin (m/v) and 5% (vol/vol) polyethylene sorbitol ester (Tween 80) in sterile Dulbecco’s PBS (calcium chloride/magnesium chloride-free, Gibco). After 1 h at 37 °C, fluorescence was determined at 530 nm excitation and 590 nm emission (SpectraMax M5, Molecular Devices).

### Colony-Forming Unit (CFU) Assay

R and NR assays were set up as describe above. CFU assays used single-cell suspensions of Mtb prepared by centrifuging cells at 123*g* for 10 min with no brake. Test agents were added once at time zero or daily for the first 5 days. Bacilli were enumerated on Middlebrook 7H11-OADC plates and incubated at 37 °C at 20% O_2_ and 5% CO_2_ for 3–4 weeks.

### Solubility Testing

Compounds were prepared at 1 mg/mL and left overnight at room temperature with stirring in phosphate buffer, 50 mM, at pH 7.4 or in 0.1 N HCl at pH 1. After separation of the soluble fraction by centrifugation, samples were filtered onto a polytetrafluoroethylene 45 μM filter (Millipore) and then analyzed by HPLC.

### hERG Channel Measurements

The potency of the compounds in inhibiting the human ERG potassium channel (*h*ERG) tail current was assessed in a recombinant HEK293 cell line stably transfected with *h*ERG cDNA under an inducible promoter using the rapid ICE (rapid ion channel electrophysiology) assay. Rapid ICE is an automated patch clamp assay utilizing a QPatch HTX system (Sophion Bioscience A/S). Briefly, inducible HEK *h*ERG cells were cultivated in minimum essential medium supplemented with 10% FBS, 1% nonessential amino acids, 1% sodium pyruvate, 2 mM l-glutamine, 15 μg/mL blasticidin, and 100 μg/mL hygromycin. *h*ERG channel induction was obtained by adding 10 μg/mL tetracycline for 24, 48, or 72 h before recordings. On the day of the experiment, cells were detached with TrypLE and prepared to be loaded on the instrument. Cells were resuspended in 7 mL of serum-free media containing 25 mM Hepes and soybean trypsin inhibitor and immediately placed in the cell storage tank of the machine. The composition of the extracellular buffer was (mM) NaCl 137, KCl 4, CaCl_2_ 1.8, MgCl_2_ 1, *d*-glucose 10, and *N*-2-hydroxyethylpiperazine-*N*′-2-ethanesulfonic acid (HEPES) 10 and adjusted to pH 7.4 with 1 M NaOH. The composition of the intracellular solution was (mM) KCl 130, MgCl_2_ 1, ethylene glycol-bis(β-aminoethyl ether)-*N*,*N*,*N*′,*N*′-tetraacetic acid (EGTA) 5, MgATP 5, and HEPES 10 and adjusted to pH 7.2 with 1 M KOH. The voltage protocol included the following steps: step from −80 to −50 mV for 200 ms, +20 mV for 4.8 s, step to −50 mV for 5 s, and then step to the holding potential of −80 mV. Compounds were dissolved in DMSO and diluted in extracellular buffer to achieve final test concentrations (3, 10, and 30 μM) in 0.3% DMSO. The voltage protocol was run and recorded continuously during the experiment. The vehicle, corresponding to 0.3% DMSO in extracellular buffer, was then applied for 3 min, followed by the test substance in triplicate. The standard combined exposure time was 5 min. The average of tail current amplitude values recorded from four sequential voltage pulses was used to calculate for each cell the effect of the test substance by calculating the residual current (% control) compared with vehicle pretreatment. Data were reported as % inhibition for each concentration tested, and IC_50_ values were estimated using QPatch software. At least three cells were tested.

### Metabolic Stability in Human Liver Microsomes and P450 Induction Studies Using Primary Human Hepatocytes

Metabolic stability and induction of human hepatocytes were performed as previously described.^71^

### In Vitro DMPK

All assays were carried out in duplicate by WuXi in Beijing, China. In addition to specific controls for each assay, meropenem and ertapenem were also included as controls in the following assays:

1. Stability in SGF and fasted simulated intestinal fluid (FaSSIF). The stability of 10 μM compounds was determined in SGF at a pH of 1.2 and fasted simulated intestinal fluid (FaSSIF) at a pH of 6.5 for 24 h at room temperature. At 0–24 h, an aliquot of the incubation mixture was removed and mixed with acetonitrile spiked with an internal standard. Samples were analyzed by LC/MS/MS, and disappearance of test compounds was assessed based on peak height and the ratio of the analyte to the internal standard. Omeprazole and chlorambucil were included as positive controls for SGF and FaSSIF stability, respectively.
2. In vitro stability in CD-1 mouse, SD rat, beagle dog, cynomolgus monkey, and human plasma. The test compound at 2 μM was incubated with male CD1 mouse, SD rat, beagle dog, cynomolgus monkey, and human plasma at 37 °C for 2 h. At 0, 10, 30, 60, and 120 min, an aliquot of the incubation mixture was removed and mixed with organic solvent spiked with the internal standard to stop the reaction. Samples were analyzed by LC/MS/MS, and disappearance of test compounds assessed based on peak height and ratios of the analyte to the internal standard. The following positive controls were used: eucatropine and/or propantheline (for mouse, monkey, and human), enalapril (for rat), and bisacodyl (for dog).
3. In vitro investigation of the passive membrane permeability/efflux transporter substrate in human Caco-2 cells at pH = 7.4. The passive cellular permeability (Papp) was determined using Caco-2 cells from ATCC. Transport of the test agent at 3 μM was measured in two directions (apical to basolateral [AB] and basolateral to apical [BA]) in DMEM buffer at pH 7.4 at 120 min incubation time, with and without the presence of 2 μM elacridar, a *P*-glycoprotein (*P*-gp) inhibitor. Exact permeability and mass balance were calculated in both directions. Amprenavir (a *P*-gp substrate) and propranolol (a highly permeable compound) were included as reference compounds. Samples (dosing solution, donor solution, and receiver solution) were analyzed by LC–MS/MS to measure concentrations of test agents and reference compounds. The compound concentrations were expressed as an area ratio determined by dividing the analyte peak area by the internal standard peak area. Transepithelial/transendothelial electrical resistance (TEER) measurements were performed in each well at the beginning of the experiment. At the end of permeability experiments, the integrity of the cell monolayers was evaluated using the paracellular permeability marker Lucifer yellow (LY) in the apical to basolateral direction in each well.
4. Plasma protein binding. *In vitro* plasma protein binding of test compounds was measured in plasma from mouse, rat, dog, monkey, and human using equilibrium dialysis at a nominal concentration of 2 μM. The unbound fraction and percentage of recovery were estimated for each of the compounds in the different matrix. The RED inserts were placed in the 48 wells of the Teflon plate (Pierce). Samples were prepared by mixing test compounds with plasma at the appropriate concentrations to yield a final drug concentration of 2 μM. Triplicate aliquots of plasma containing test compounds were pipetted to the plasma side of the insert, and PBS (phosphate-buffered saline) at pH 7.4 was placed into the receiver side of the insert. The plate was covered with sealing tape and incubated in a 37 °C orbital shaker–water bath at approximately 150 rpm for 4 h. Following incubation, samples were prepared in a mixed matrix configuration. Aliquots of samples were pipetted into 96-well plates, and precipitation buffer was added to precipitate the protein in the samples. Samples were then mixed by vortexing and centrifuged, and the supernatant was assayed directly by LC/MS/MS.
5. Mouse PK studies. (i) Animals and ethics assurance. Animal studies were carried out in accordance with the Guide for the Care and Use of Laboratory Animals,^72^ with approval from the Institutional Animal Care and Use Committee (IACUC) of the New Jersey Medical School, Rutgers University, Newark, NJ. All animals were maintained under specific pathogen-free conditions and fed water and chow ad libitum, and all efforts were made to minimize suffering or discomfort. Compounds TBBL-0000001, TBBL-0000005, TBBL-0000009, TBBL-0000012, TBBL-0000310, TBBL-0000316, and TBBL-0000552 were subjected to in vivo PK profiling in mice. In intravenous (i.v.) PK studies, two female CD-1 mice received a single 5 mg/kg dose of the experimental compound prepared in 5% DMA/95% [4% Cremophor] and injected via the tail vein. Blood samples were collected in K_2_EDTA-coated tubes predose and 1, 15 min, 1, 3, and 5 h postdose from each mouse via microsampling through the tip of the tail vein. In oral (p.o.) PK studies, two female CD-1 mice received a single 25 mg/kg dose of the experimental compound prepared in 5% DMA/60% PEG300 in D5W (5% dextrose in water). Blood samples were collected in K_2_EDTA-coated tubes predose and 0.5, 1, 3, and 5 h postdose. Blood was kept on ice and centrifuged to recover plasma, which was stored at −80 °C until analysis by high-pressure liquid chromatography coupled to tandem mass spectrometry (LC–MS/MS). (ii) LC–MS/MS analytical methods. LC–MS/MS analysis was performed on a Sciex Applied Biosystems Qtrap 6500+ triple-quadrupole mass spectrometer coupled to a Shimadzu Nexera 2 HPLC system to quantify each drug in plasma. Neat 1 mg/mL DMSO stocks for each compound were serially diluted in 50/50 acetonitrile water to create standard curves and QC spiking solutions. Standards and QC (quality control samples) were created by adding 10 μL of spiking solutions to 90 μL of drug-free plasma (CD-1 K_2_EDTA Mouse, Bioreclamation IVT). A total of 20 μL of control, standard, QC, or study sample plasma was added to 200 μL of acetonitrile/methanol 50/50 protein precipitation solvent containing the internal standard (10 ng/mL Verapamil). Extracts were vortexed for 5 min and centrifuged at 4000 rpm for 5 min A total of 100 μL of supernatant was transferred for HPLC-MS/MS analysis and diluted with 100 μL of Milli-Q deionized water. Chromatography was performed on an Agilent Zorbax SB-C8 column (2.1 × 30 mm; particle size, 3.5 μm) using a reverse-phase gradient. Milli-Q deionized water with 0.1% formic acid was used for the aqueous mobile phase and 0.1% formic acid in acetonitrile for the organic mobile phase. Multiple-reaction monitoring of precursor/product transitions in the electrospray positive-ionization mode was used to quantify the analytes. Sample analysis was accepted if the concentrations of the QC samples were within 20% of the nominal concentration. Data processing was performed using Analyst software (version 1.6.2; Applied Biosystems Sciex).

### Treatment of Dehydropeptidase-1 (DHP-1) Knockout Mice

After a 1-week acclimation period, pathogen-free, 8–10 weeks old female 129sv DHP-1 knockout mice (Taconic DK) were intratracheally infected with 10^5^ CFU of wild-type Mtb H37Rv. From days 9–16 after infection, mice received subcutaneous dosing (b.i.d. for days 9–14, q.d. for d15) of test agents and oral dosing of clavulanate. Meropenem and clavulanate stocks were prepared daily, and other β-lactams were prepared every 72 h. Meropenem was dosed at 50 and 300 mg/kg sc, clavulanate was dosed 100 mg/kg po, and test agents were dosed at 50 mg/kg sc. To plate for CFU, lung homogenates were thawed, and bacilli were enumerated on Middlebrook 7H11 solid medium supplemented with 10% OADC and 0.4% activated charcoal for ∼18 days at 37 °C. 20 μL blood samples were taken from the lateral tail vein at target times of 0.5, 1, 3, and 6 h to measure blood concentrations of β-lactams. Healthy mice were sacrificed on day 16, and mice that displayed severe weight loss (>20%) were sacrificed at earlier time points. Lung lobes were aseptically removed, homogenized, and frozen. These animal studies were ethically reviewed and carried out in accordance with the European Directive 2010/63/EEC and the GSK policy on the Care, Welfare, and Treatment of Animals.

### Clustering of Replicating-Active β-Lactams

β-Lactams were clustered according to their structural features using Canvas (Version 3.6.013) in the Schrodinger suite (2018 version). The binary fingerprints were used to represent a chemical structure, and a hierarchical clustering method was applied to generate 19 clusters.^73^

### Analysis of >600 Shared β-Lactam Structures

The degree of library overlap between the four pharma collections was determined using a KNIME workflow named AMG_File_Comparison written by Mark Gardner of AMG consultants (https://www.mmv.org/research-development/computational-chemistry/global-health-compound-design-webinars; slide deck, “a simple KNIME script to compare compound collections”). The protocol allows for direct comparison of two chemical structure sets and can be adjusted to return both exact matches and similar matches. In the second case, compounds with a similarity of >0.85 (Tanimoto) were considered matches. Consecutive head-to-head comparisons were used to populate the binary overlap portions of both the exact and similar Venn diagrams. Population of the tertiary and quaternary portions of the exact Venn diagram was accomplished by creating and then searching against combined sets.

## Supporting Information Available

Replicating and nonreplicating activity of select β-lactams at the cherry-picking stage; screenshot of the TBDA β-lactam vault within CDD; structural overlap among β-lactams of pharma collections; distribution of chemical properties; microbial spectrum and human hepatoma cell (HepG2) toxicity of replicating- and dual-active β-lactams; and plasma pharmacokinetics of β-lactams in uninfected CD-1 mice and Mtb-infected DHP-1 knockout mice; summary of primary screening; summary of dose–response cherry picking under replicating and nonreplicating conditions; summary of clavulanate dependence of β-lactam activity against replicating Mtb Δ*panCD*Δ*lysA*; clustering and characterization of replicating-active beta-lactams; summary of β-lactam DMPK; summary of β-lactam pharmacokinetics in uninfected CD-1 mice; summary of β-lactam pharmacokinetics after subcutaneous dosing of uninfected DHP-1 mice; summary of β-lactam pharmacokinetics in Mtb-infected DHP-1 knockout mice after subcutaneous administration; and summary of CFU enumerating viable *M. tuberculosis* from the lungs of untreated and β-lactam-treated DHP-1 knockout mice, including detailed descriptions of mouse health and viability (PDF)

## Notes

Stock of TBBL-0000316 and other β-lactams described in this paper will be provided on a case-by-case basis. Sharing of compounds will depend on numerous factors, including the quantity of remaining stocks and the purity of the stock. We will otherwise attempt to share synthetic routes.

## Notes

The data associated with the β-lactams with structures revealed in this article will be shared as a publicly available, free database within CDD at www.collaborativedrug.org.
