# Supplementary material for: Identification of β-Lactams Active against Mycobacterium tuberculosis by a Consortium of Pharmaceutical Companies and Academic Institutions
Source: ACS Infect Dis. 2022 Feb 22;8(3):557–73. doi: 10.1021/acsinfecdis.1c00570 (PMC8922279; doi:10.1021/acsinfecdis.1c00570)
Supplement: Supplementary file 1 — id1c00570_si_001.pdf [file id1c00570_si_001.pdf]

Supporting Information

Identification of β-lactams active against *Mycobacterium tuberculosis* by a consortium of pharmaceutical companies and academic institutions

One sentence summary: *A consortium of pharmaceutical companies and academic institutions collaborated to identify β-lactam antibiotics active against* Mycobacterium tuberculosis.

Ben Gold[1*], Jun Zhang[1], Landys Lopez Quezada[1], Julia Roberts[1], Yan Ling[1], Madeleine Wood[1], Wasima Shinwari[1], Laurent Goullieux[2a,3], Christine Roubert[2a,3], Laurent Fraisse[2a,€], Eric Bacqué[2a,3], Sophie Lagrange[2a,3], Bruno Filoche-Rommé[2b], Michal Vieth[4a], Philip A. Hipskind[4b‡], Louis N. Jungheim[5], Jeffrey Aubé[6], Sarah M. Scarry[6], Stacey L. McDonald[6], Kelin Li[6], Andrew Perkowski[6], Quyen Nguyen[6], Véronique Dartois[7 ¥], Matthew Zimmerman[7 ¥], David B. Olsen[8], Katherine Young[8], Shilah Bonnett[9], Douglas Joerss[9+], Tanya Parish[9+], Helena I. Boshoff[10], Kriti Arora[10%], Clifton E. Barry III[10], Laura Guijarro[11,] Sara Anca[11,] Joaquín Rullas[11], Beatriz Rodríguez-Salguero[11], Maria S. Martínez-Martínez[11], Esther Porras-De Francisco[11], Monica Cacho[11], David Barros-Aguirre[11], Paul Smith[12], Steven J. Berthel[13], Carl Nathan[1*], Robert H. Bates[11*]

[1] Department of Microbiology & Immunology, Weill Cornell Medicine, 413 East 69th Street, New York, NY, 10021, USA
[2a] Sanofi, Infectious Diseases Therapeutic Area, 69280 Marcy l'Étoile, France
[2b] Sanofi, Integrated Drug Discovery, 94400 Vitry sur Seine, France
[3] Evotec (Lyon), 69007 Lyon, France
[4a] Lilly Biotechnology Center, Eli Lilly and Company, 10290 Campus Point Dr, San Diego, CA 92121, USA
[4b] Eli Lilly and Company, Lilly Research Laboratories, Lilly Corporate Center, Indianapolis, IN 46285, USA
[5] YourEncore, 20 North Meridian Street, Indianapolis, IN, 46204, USA
[6] Division of Chemical Biology and Medicinal Chemistry, UNC Eshelman School of Pharmacy, University of North Carolina, Chapel Hill, NC, 27599, USA
[7] Public Health Research Institute, New Jersey Medical School, Rutgers, The State University of New Jersey, Newark, NJ, 07103, USA
[8] Merck & Co., Inc., Infectious Diseases, 770 Sumneytown Pike, West Point, PA, 19486, USA
[9] TB Discovery Research, Infectious Disease Research Institute, 1616 Eastlake Ave E, Suite 400, Seattle, WA, 98102, USA
[10] Tuberculosis Research Section, Laboratory of Clinical Immunology and Microbiology, Bethesda, MD, 20892, USA

[11] Global Health Pharma R&D, GlaxoSmithKline, Severo Ochoa 2, Tres Cantos 28760, Madrid, Spain
[12] Independent Consultant, Global Health Pharma R&D, GlaxoSmithKline, Severo Ochoa 2, Tres Cantos 28760, Madrid, Spain
[13] Panorama Global, 2101 4th Avenue, Suite 2100, Seattle, WA, 98121, USA

[€] Present address: DNDi, Chemin Louis-Dunant 15, 1202 Geneva, Switzerland
[‡] Present address: Lgenia Inc., Fortville, IN, 46040, USA
[¥] Present address: Center for Discovery and Innovation, and Hackensack School of Medicine, Hackensack Meridian Health, Nutley, NJ, 07110, USA
[+] Present address: Seattle Children's Research Institute, 307 Westlake Avenue N, Seattle, WA, 98109, USA
[%]Present address: Bill & Melinda Gates Medical Research Institute, 750 Republican St, Suite F309, Seattle, WA, 98109, USA

* To whom correspondence should be addressed: E-mail: robert.h.bates@gsk.com, bsg2001@med.cornell.edu, cnathan@med.cornell.edu

RB: Global Health Pharma R&D, GlaxoSmithKline, Severo Ochoa 2, Tres Cantos 28760, Madrid, Spain; Phone: +(34) 650395529

BG: Department of Microbiology and Immunology, Weill Cornell Medicine, Belfer 1126, 413 East 69th Street, NY, NY 10021; Phone: 646-962-6209

CN: Department of Microbiology and Immunology, Weill Cornell Medicine, Belfer 1124, 413 East 69th Street, NY, NY 10021; Phone: 212-746-6505

Keywords: *Mycobacterium tuberculosis, tuberculosis, β-lactam, clavulanate, high-throughput screening, consortium*

SUPPLEMENTARY MATERIALS

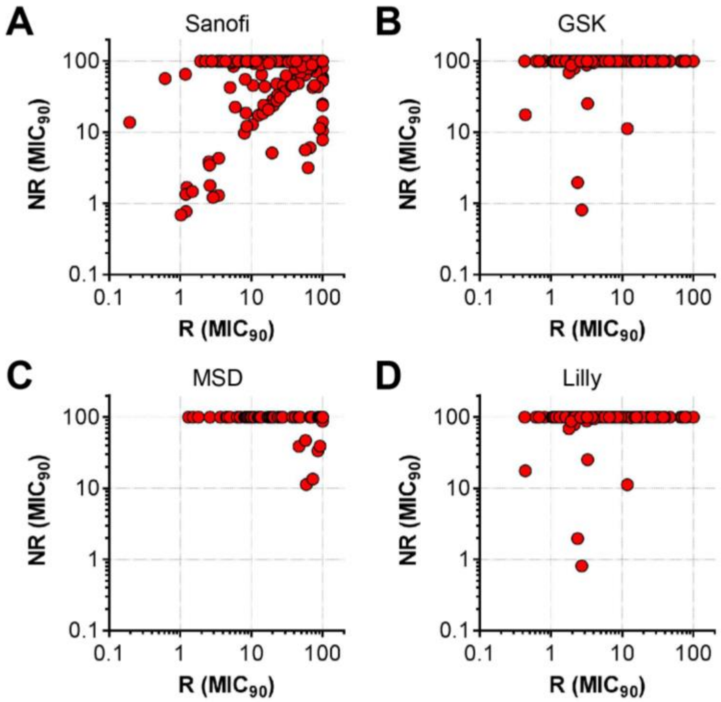

Fig. S1. Replicating (R) and nonreplicating (NR) activity of select β-lactams at the cherry-picking stage of screening. For a select set of compound chosen for further study, the charcoal agar resazurin assay (CARA) demonstrated that the NR activity of most β-lactams, with the exception of cepalosporins bearing a 2-mercaptopyridine N-oxide [1], was almost always attributed to compound carry-over during the replicating recovery phase of the NR assay [2].

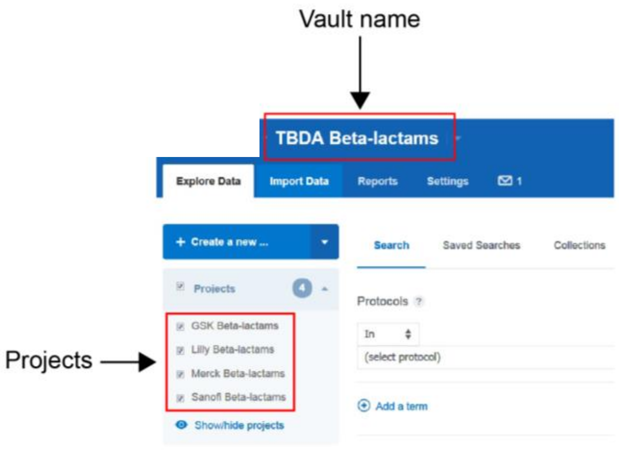

Fig. S2. Screenshot of the TBDA β-lactam vault within Collaborative Drug Discovery (CDD). The "vault name" (currently illustrating "TBDA β-lactams") is an option from a pull-down menu that permits changing vaults. A project can be activated or deactivated by checking or unchecking the box on the left of each project name. By employing tiered access to CDD Vault, members can only gain access to projects for which access was approved by the TBDA medicinal chemist and/or data coordinator.

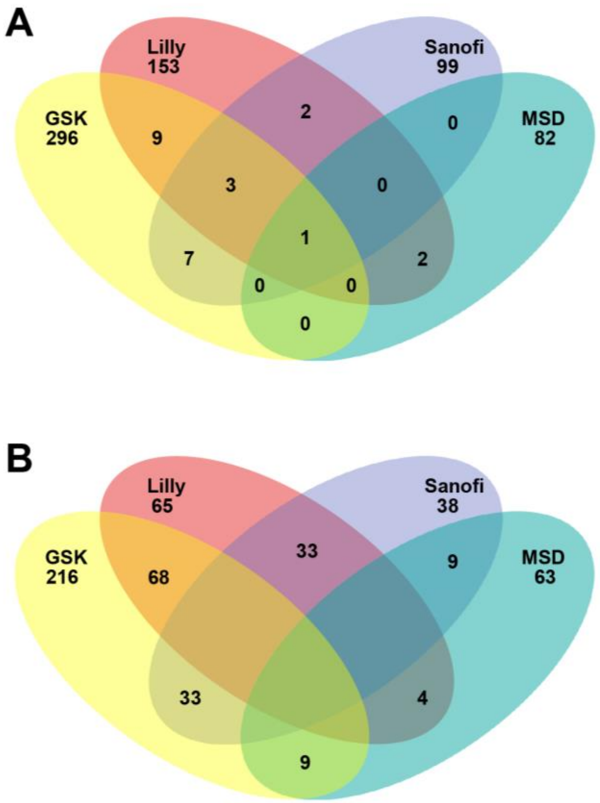

Fig. S3. Surprisingly low structural overlap among β-lactams of pharma collections. Venn-diagram analysis of β-lactams from GSK, Lilly, Sanofi and MSD whose structures are (**a**) identical or (**b**) similar using Tanimoto identifiers. The degree of library overlap between the four pharma collections was determined using a KNIME protocol named AMG_File_Comparison written by Mark Gardner of AMG consultants. The protocol allows for direct comparison of two chemical structure sets and can be adjusted to return both exact matches as well as similar matches. In this case, compounds with a similarity of >0.85 (Tanimoto) were considered matches. Consecutive head-to-head comparisons were used to populate the binary overlap portions of both the exact and similar Venn diagrams. Population of the tertiary and quaternary portions of the exact Venn diagram was accomplished by creating and searching against combined sets.

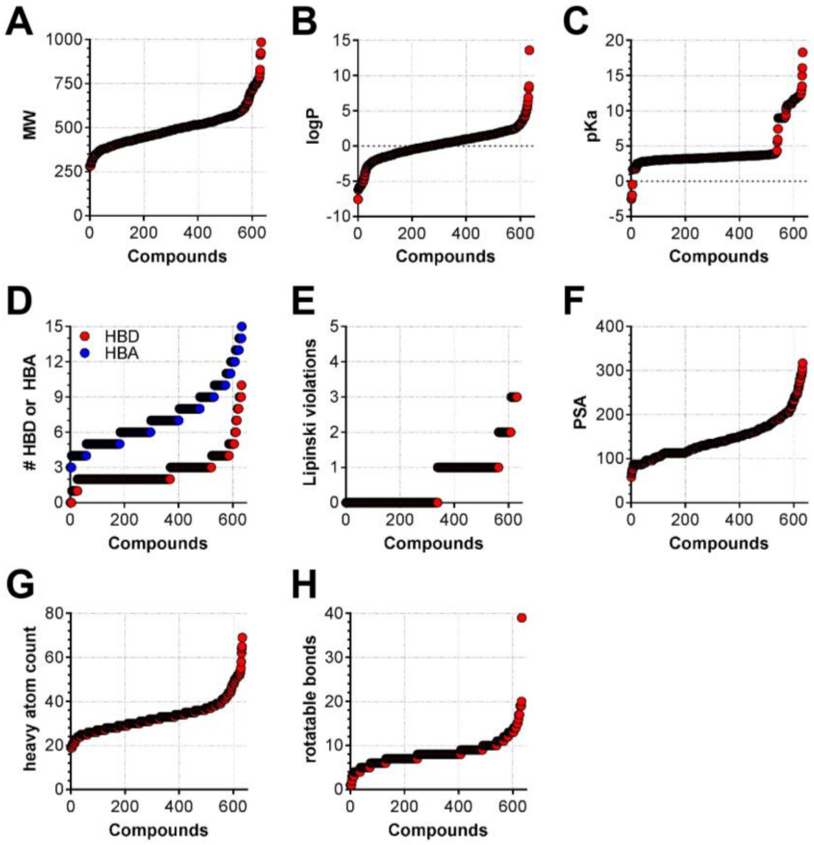

Fig. S4. Distribution of chemical properties. Chemical properties of β-lactams whose structures were revealed by GSK, Lilly, Sanofi and MSD were calculated with ChemAxon software within CDD Vault.

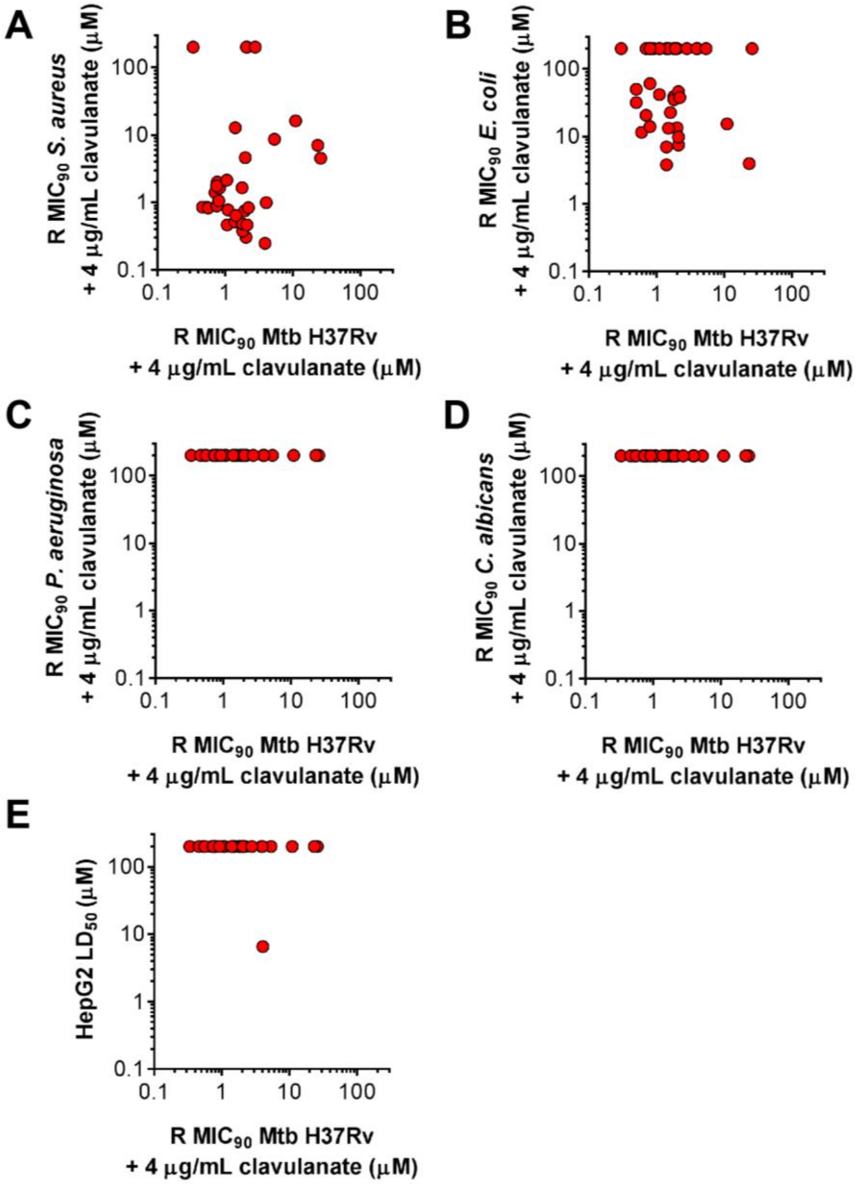

Fig. S5. Microbial spectrum and human hepatoma cell (HepG2) toxicity of R-active β-lactams. The same data is shown in **Supplementary Table S4**.

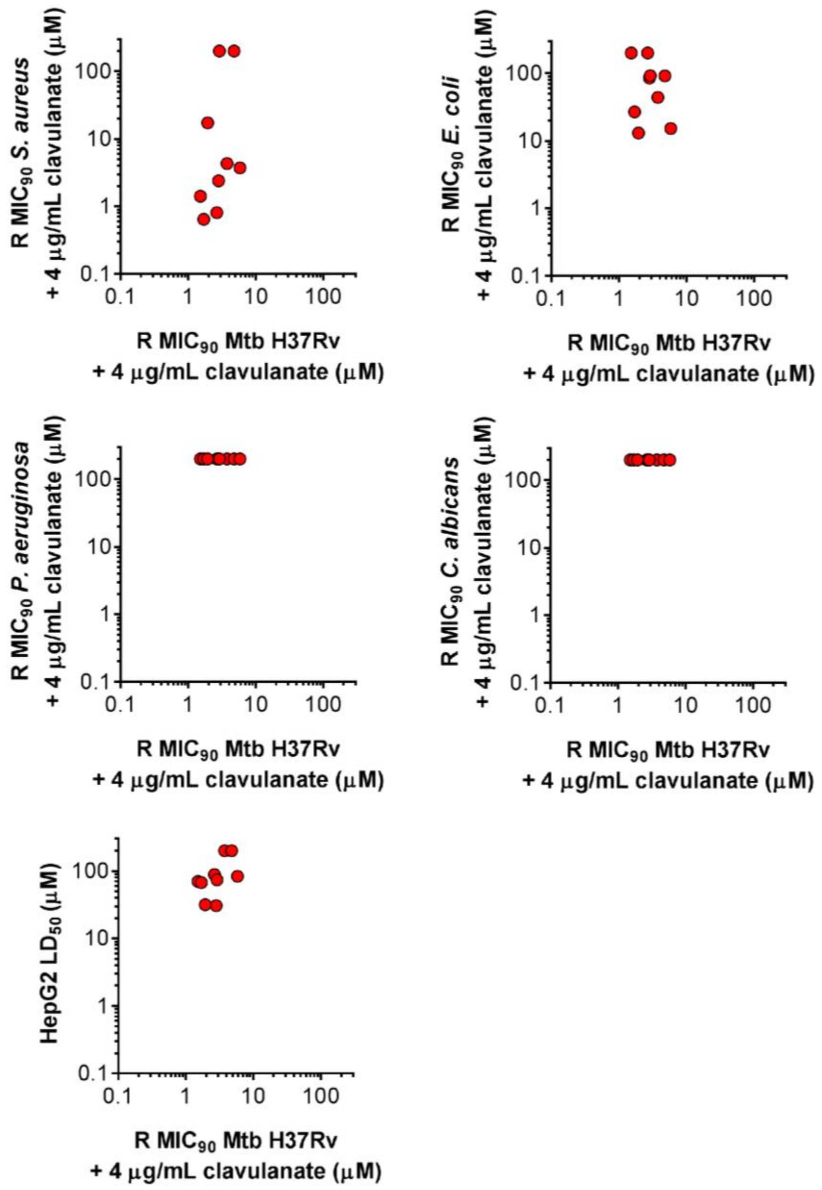

Fig. S6. Microbial spectrum and human hepatoma cell (HepG2) toxicity of candidate dual-active β-lactams. The candidacy of compounds as dual-actives was based on calculated MIC_{90} values in liquid broth medium ($0.2 \leq [\text{R-MIC}_{90} / \text{NR-MIC}_{90}] \leq 5$ [2]). The data is the same as in **Supplementary Table S4**.

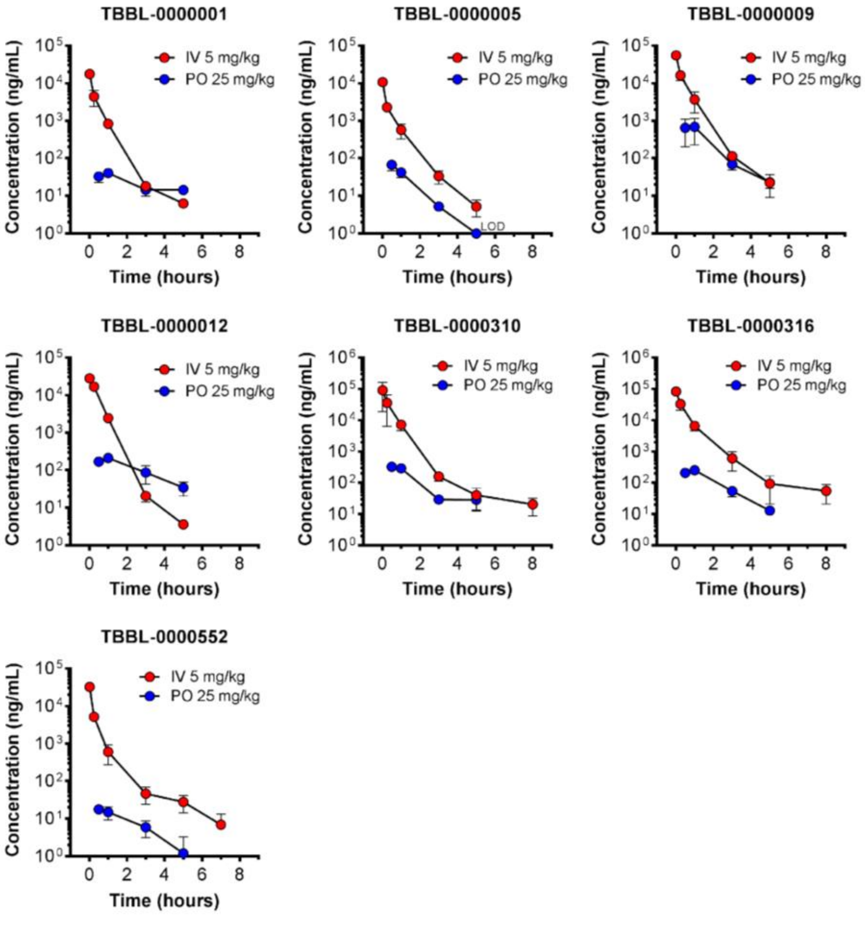

Fig. S7. Plasma pharmacokinetics of β-lactams in uninfected CD-1 mice. Mice were dosed intravenously (IV) (5 mg/kg) or per oral (PO) (25 mg/kg). Data points represent the mean plus and minus the standard deviation of three mice.

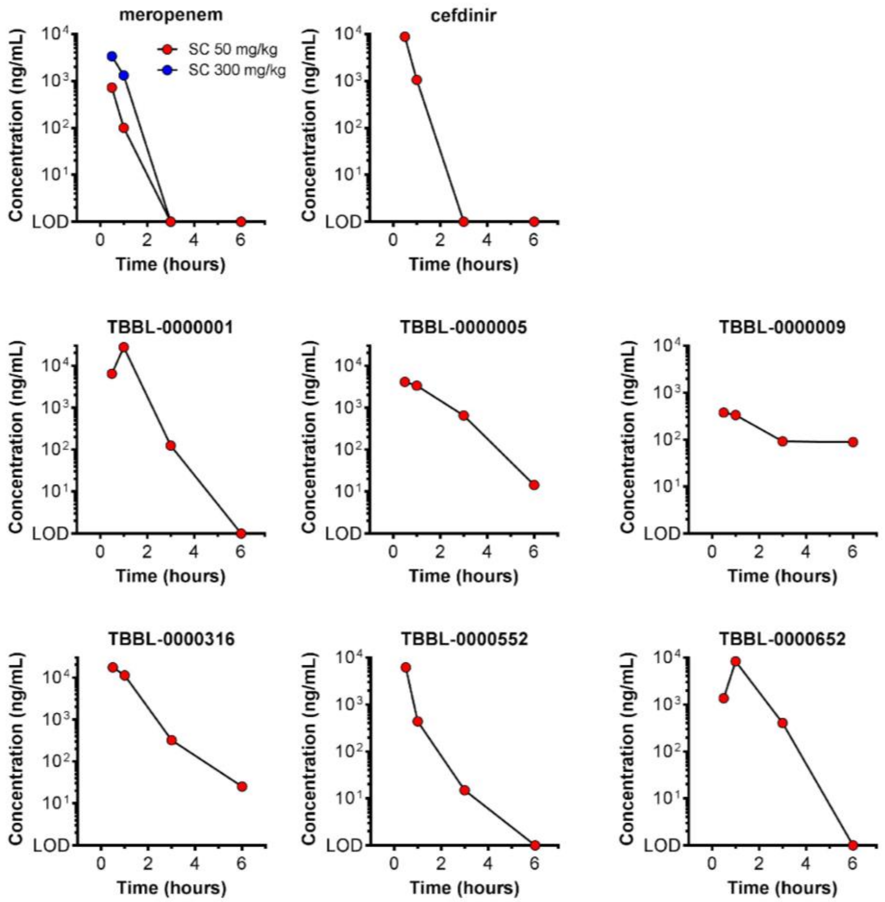

Fig. S8. Plasma pharmacokinetics of β-lactams in Mtb-infected DHP-1 knockout mice. These data correspond to **Figure 6b.** β-lactams were SC dosed BID at 50 mg/kg and PO dosed BID with 100 mg/kg clavulanate. Meropenem was dosed at both 50 and 300 mg/kg BID with 100 mg/kg clavulanate. To graph the data, the limit of detection (LOD) was set at 1 ng/mL

Table S1. Summary of primary screening.

	Screening condition		Source of compounds				
**Screening criteria**	**R + clav (% inhibition)**	**NR (% inhibition)**	**Sanofi**	**GSK** [a]	**Lilly** [b, c]	**MSD** [b, d]	**Total**
**Total β-lactams supplied**			5716	566 [a]	2153	426	8861
**R active, stringent**	≥ 90%	≤ 50%	898 (15.7%)	170 (30.0%)	516 (24.0%)	14 (3.3%)	1598 (18.0%)
**R active**	≥ 80%	≤ 50%	1115 (19.5%)	205 (36.2%)	642 (29.8%)	32 (7.5%)	1994 (22.5%)
**NR active**	≤ 50%	≥ 80%	129 (2.3%)	3 (0.5%)	N.D.	N.D.	132 (1.5%)
**Dual active**	≥ 80%	≥ 80%	120 (2.1%)	15 (2.7%)	N.D.	N.D.	135 (1.5%)

clav, clavulanate at 4 μg/mL

[a], Includes 33 molecules screened in a previous study [1].

[b], MSD-NIAID and Lilly-IDRI did not test activity under NR conditions for the primary HTS.

[c], Lilly-IDRI re-tested 511 active β-lactams +/- clavulanate before sending compounds to WCM.

[d], MSD-NIAID set up the primary HTS in dose-response format in the presence and absence of 4 μg/mL clavulanate.

Table S2. Summary of dose-response assays of prioritized β-lactams at WCM under both replicating and nonreplicating conditions.

		WCM - MIC_{90} (μM)						
		Screening condition		Source of compounds				
**Screening criteria**	**Criteria used to make activity prediction [2]**	**R + clav**	**NR**	**Sanofi**	**GSK [a]**	**Lilly [a]**	**MSD [a]**	**Total**
**Total supplied**				1421	304	163	80	1968
**R active**		≤ 20		215	110	138	29	492
**NR active**	if R-MIC_{90} ≤ NR-MIC_{90}, then [(R-MIC_{90})/(NR-MIC_{90})] ≥ 5		≤ 20	0	5	0	2	7
**candidate dual active**	0.2 ≤ [(R-MIC_{90})/(NR-MIC_{90})] ≤ 5	≤ 20	≤ 20	16	17	3	0	36
**completely inactive**	< 50% inhibition at 100 μM	> 100	> 100	180	8	0	26	214

clav, clavulanate at 4 μg/mL

[a], The MIC_{90} of some molecules was determined at IDRI, NIAID, or GSK in prior to shipping to WCM.

Table S3. Clavulanate-dependence of β-lactam activity against replicating Mtb Δ*panCD*Δ*lysA*.

	WCM - MIC90 (μM)					
	**Screening condition**	**Source of compounds**				
**Screening criteria**	**R + clav**	**Sanofi**	**GSK**	**Lilly**	**MSD**	**Total**
**Total supplied**		118	59	159	80	416
**R active**	≤ 20	112	55	114	26	307
**R active with ≥ 4-fold MIC_{90} shift +/- clavulanate**	≤ 20	99	35	114	23	271
**% clavulanate-dependent**		88.4%	63.6%	100.0%	88.5%	88.3%

clav, clavulanate at 4 μg/mL

Table S4. (part 1 of 4) Characterization of R-active β-lactams.

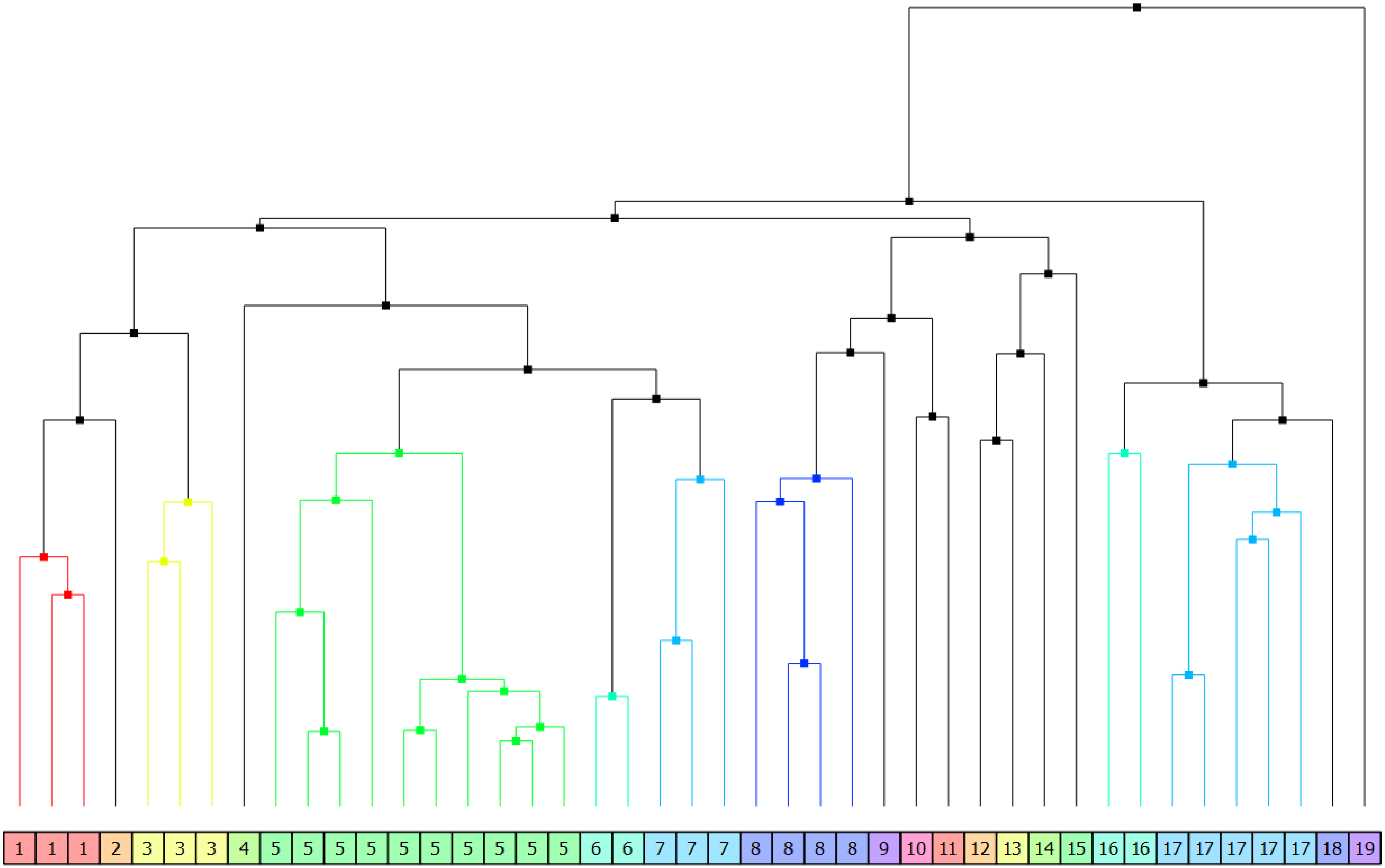
1 1 1 2 3 3 3 4 5 5 5 5 5 5 5 5 5 5 6 6 7 7 7 8 8 8 8 9 10 11 12 13 14 15 16 16 17 17 17 17 17 18 19

Table S4. (part 2 of 4) Characterization of R-active β-lactams.

* All values in μM. Clavulanate used at 4 μg/mL.

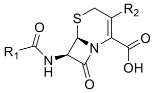

X1

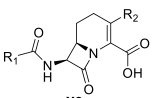

X2

Name	Structure			Structural Cluster	Mtb H37Rv				*E.coli*	*P.aeruginosa*	*S.aureus*	*C.albicans*	
	R1	R2	X		R	R + CLAV	NR	NR + CLAV	R + CLAV	R + CLAV	R + CLAV	R	HepG2 LD50
**TBBL-0000418**	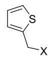	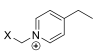	X1	1	100.0	1.4	> 100	> 100	7.0	> 100	0.5	> 100	> 100
**TBBL-0000466**	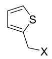	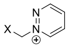	X1	1	39.0	1.8	> 100	> 100	35.1	> 100	0.5	> 100	> 100
**TBBL-0000493**	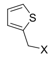	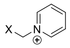	X1	1	50.0	2.2	> 100	> 100	37.9	> 100	0.8	> 100	> 100
**TBBL-0000009**	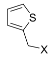	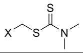	X1	2	8.0	4.0	> 100	> 100	> 100	> 100	1.0	> 100	6.6
**TBBL-0000417**	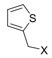	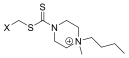	X1	3	173.0	1.8	> 200	> 100	39.2	> 200	1.7	> 200	> 100
**TBBL-0000419**	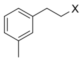	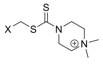	X1	3	43.1	0.7	> 200	> 100	> 200	> 200	1.4	> 200	> 100
**TBBL-0000421**	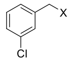	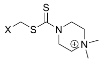	X1	3	83.1	2.0	> 200	> 100	> 200	> 200	0.3	> 200	> 100
**TBBL-0000330**	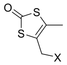	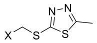	X1	4	27.6	2.8	> 100	> 100	> 100	> 100	> 100	> 100	> 100
**TBBL-0000309**	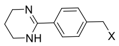	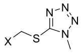	X1	5	29.6	0.7	> 100	> 100	20.6	> 100	1.8	> 100	> 100
**TBBL-0000310**	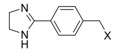	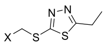	X1	5	16.4	0.5	90.4	92.2	49.9	> 100	0.9	> 100	> 100
**TBBL-0000311**	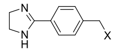	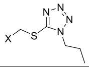	X1	5	14.1	0.8	> 100	> 100	> 100	> 100	0.9	> 100	> 100
**TBBL-0000312**	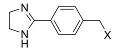	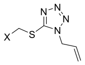	X1	5	22.1	0.8	> 100	> 100	> 100	> 100	1.1	> 100	> 100
**TBBL-0000313**	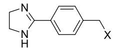	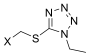	X1	5	17.3	0.6	> 100	> 100	11.6	> 100	0.8	> 100	> 100
**TBBL-0000314**	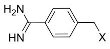	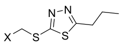	X1	5	14.1	0.8	> 100	> 100	> 100	> 100	1.6	> 100	> 100
**TBBL-0000316**	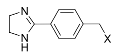	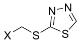	X1	5	44.7	0.5	> 100	> 100	31.8	> 100	0.9	> 100	> 100

Table S4. (part 3 of 4) Characterization of R-active BLAs.

* All values in μM. Clavulanate used at 4 μg/mL.

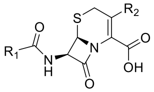

X1

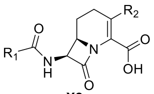

X2

Name	Structure			Structural Cluster	Mtb H37Rv				*E.coli*	*P.aeruginosa*	*S.aureus*	*C.albicans*	
	R1	R2	X		R	R + CLAV	NR	NR + CLAV	R + CLAV	R + CLAV	R + CLAV	R	HepG2 LD50
**TBBL-0000317**	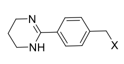	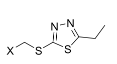	X1	5	22.3	1.1	> 100	> 100	> 100	> 100	2.1	> 100	> 100
**TBBL-0000318**	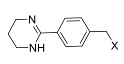	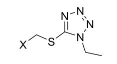	X1	5	24.5	0.8	> 100	> 100	60.5	> 100	2.0	> 100	> 100
**TBBL-0000323**	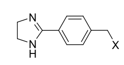	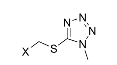	X1	5	31.9	0.8	> 100	> 100	14.0	> 100	1.8	> 100	> 100
**TBBL-0000305**	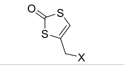	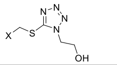	X1	6	40.7	2.1	> 100	> 100	7.5	> 100	> 100	> 100	> 100
**TBBL-0000325**	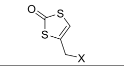	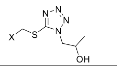	X1	6	53.2	1.4	> 100	> 100	3.8	> 100	0.6	> 100	> 100
**TBBL-0000460**	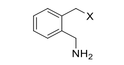	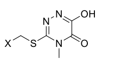	X1	7	5.4	2.0	69.3	85.6	13.4	> 200	4.6	> 200	> 100
**TBBL-0000468**	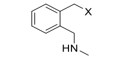	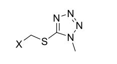	X1	7	16.4	5.4	> 100	> 100	> 100	> 100	8.7	> 100	> 100
**TBBL-0000656**	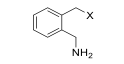	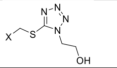	X1	7	5.8	2.1	> 100	> 100	9.8	> 100	> 100	> 100	> 100
**TBBL-0000415**	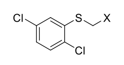	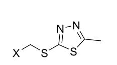	X1	8	22.9	1.6	> 100	> 100	22.7	> 100	< 0.1	> 100	> 100
**TBBL-0000446**	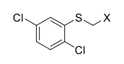	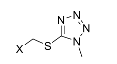	X1	8	20.7	1.5	> 100	> 100	13.3	> 100	< 0.1	> 100	> 100
**TBBL-0000467**	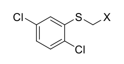	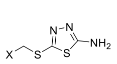	X1	8	52.2	1.5	> 100	> 100	> 100	> 100	< 0.1	> 100	> 100
**TBBL-0000497**	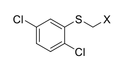	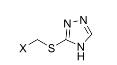	X1	8	54.1	1.8	> 100	> 100	> 100	> 100	0.4	> 100	> 100
**TBBL-0000307**	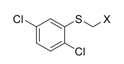	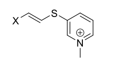	X1	9	34.8	0.9	> 100	> 100	> 100	> 100	< 0.1	> 100	> 100
**TBBL-0000306**	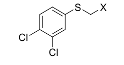	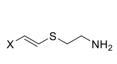	X1	10	14.4	0.3	> 100	> 100	> 100	> 100	> 100	> 100	> 100
**TBBL-0000322**	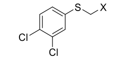	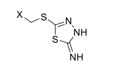	X1	11	52.3	4.0	> 100	> 100	> 100	> 100	< 0.1	> 100	> 100

Table S4. (part 4 of 4) Characterization of R-active β-lactams.

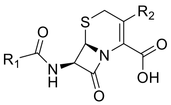
X1

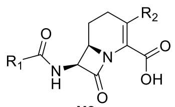
X2

* All values in µM. Clavulanate used at 4 µg/mL.

Name	Structure			Structural Cluster	Mtb H37Rv				*E.coli*	*P.aeruginosa*	*S.aureus*	*C.albicans*	HepG2 LD50
	R1	R2	X		R	R + CLAV	NR	NR + CLAV	R + CLAV	R + CLAV	R + CLAV	R	
**TBBL-0000328**	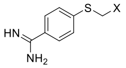	X—	X1	12	64.1	1.4	> 100	> 100	> 100	> 100	12.9	> 100	> 100
**TBBL-0000063**	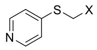	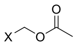	X1	13	13.1	1.9	> 100	> 100	> 100	> 100	0.8	> 100	> 100
**TBBL-0000010**	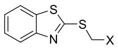	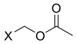	X1	14	62.0	3.9	> 100	> 100	> 100	> 100	0.2	> 100	> 100
**TBBL-0000058**	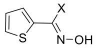	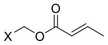	X1	15	8.7	1.1	56.8	84.6	41.8	> 100	0.5	> 100	> 100
**TBBL-0000005**	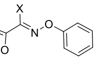	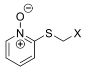	X1	16	2.9	2.7	1.8	1.5	> 100	> 100	0.8	> 100	88.7
**TBBL-0000539**	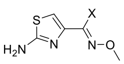	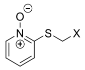	X1	16	2.0	1.9	4.7	4.6	13.1	> 100	17.3	> 100	31.7
**TBBL-0000001**	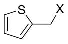	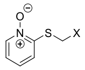	X1	17	3.0	1.7	1.8	2.2	26.9	> 100	0.6	> 100	67.5
**TBBL-0000002**	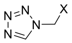	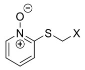	X1	17	5.7	5.8	4.4	3.0	15.3	> 100	3.7	> 100	83.5
**TBBL-0000003**	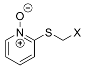	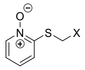	X1	17	4.8	3.8	3.2	3.5	44.1	> 100	4.3	> 100	> 100
**TBBL-0000004**	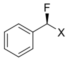	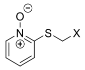	X1	17	1.2	1.5	1.7	2.3	> 100	> 100	1.4	> 100	70.2
**TBBL-0000007**	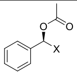	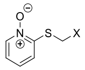	X1	17	2.3	2.8	2.3	2.5	85.1	> 100	2.4	> 100	30.7
**TBBL-0000552**	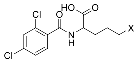	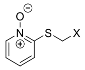	X1	18	2.8	2.9	1.9	2.1	92.6	> 100	> 100	> 100	74.3
**TBBL-0000435**	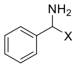	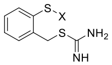	X2	19	90.2	1.1	> 100	> 100	> 100	> 100	0.8	> 100	> 100

Table S5. Summary of DMPK assays run with prioritized β-lactams and control molecules meropenem and ertapenem.

TBBL-#	Biorelevant media stability				Plasma stability						Caco-2 permeability							Plasma binding				
	FaSSIF Stability, % remaining		SGF Stability, % remaining		Time Point (min)	% Remaining					Mean P_{app} (10^{-6} cm/s)		Efflux Ratio	Mean Recovery %		Rank		% bound to plasma				
	6h	24h	1h	24h		Mouse	Rat	Dog	Monkey	Human	A to B	B to A		A to B	B to A	P_{app}	Efflux Transporter Substrate	Cynomolgus Monkey	Beagle Dog	CD-1 Mouse	SD Rat	Human
**TBBL-0000001**	`	113.92	102.04	115.87	120	79.6	80.6	54.7	117.1	77.3	<0.04	<0.03	NA	<93.09	<99.80	Low	NA	83.4	47.1	62.6	63.1	78.0
**TBBL-0000058**	114.21	66.32	94.94	57.36	120	67.6	60.9	81.2	22.0	35.0	<0.38	0.63	>1.68	<98.97	111.16	Low	Poor or no					
**TBBL-0000005**	98.71	98.47	120.19	76.59	120	60.6	35.2	51.3	107.8	73.7	<0.33	<0.28	NA	<88.88	<89.82	Low	NA	94.0	81.4	94.0	87.9*	87.1
**TBBL-0000004**	88.58	94.96	71.91?	141.71?	120	84.1	71.0	64.1	103.9	81.0	<0.12	<0.11	NA	<98.09	<112.70	Low	NA	78.2	59.2	65.5	62.0	74.1
**TBBL-0000009**	85.28	90.87	31.69	9.96	120	93.0	95.8	104.2	132.7	97.9	<0.50	<0.43	NA	<98.33	<102.86	Moderate	NA	95.3	91.6	90.5	97.8	96.6
**TBBL-0000063**	102.47	62	87.31	27.14							<0.41	<0.37	NA	<87.73	<102.87	Low	NA	0.0	27.8	14.7	42.4	38.7
**Meropenem**	94.11	76.75	110.54	100.24	120	74.7	57.7	75.2	99.9	70.6	<0.26	<0.24	NA	<90.96	<94.15	Low	NA					
**TBBL-0000018**	89.36	36.86	95.78	0	120	0.0	0.0	0.0	0.0	0.0								NA, low PS	NA, low PS	NA, low PS	NA, low PS	NA, low PS
**TBBL-0000012**	120.19	112.36	91.41	85.76	120	84.5	82.3	83.2	111.7	78.1	<0.10	0.23	>2.21	<97.40	102.98	Low	Likely	41.1	82.5	26.6	47.2	21.5
**Ertapenem**	86.76	85.75	0	0	120	74.2	69.1	70.7	114.9	77.5	<0.23	<0.21	NA	<116.22	<112.44	Low	NA					
**TBBL-0000656**	89.32	70.9	87.12	53.93	120	95.0	85.1	74.7	85.7	75.1	<0.12	0.27	>2.26	<102.47	106.09	Low	Likely	22.0	11.2	23.0	50.2	23.3
**TBBL-0000460**	96.2	86.61	102.88	85.68	120	88.8	92.4	73.7	25.6	90.4	0.16	0.24	1.52	97.79	106.35	Low	Poor or no	NA	0.0	16.1	29.0	15.9
**TBBL-0000309**	95.78	77.15	103.51	60.13	120	68.8	79.8	77.8	104.7	79.0	0.18	0.38	2.12	99.65	104.93	Low	Likely	29.4	0.0	4.1	38.2	24.6

**TBBL-0000310**	98.8	95.07	86.27	0.61	120	82.2	80.0	79.6	110.3	78.9	0.46	0.43	0.94	99.13	102.93	Low	Poor or no	56.2	30.5	49.9	81.2	60.7
**TBBL-0000316**	94.71	84.15	83.73	0.45	120	88.5	85.9	77.3	114.0	86.1	0.31	0.32	1.06	103.90	110.25	Low	Poor or no	66.5	2.7	18.3	62.2	38.1
**TBBL-0000314**	101.85	95.17	76.41	1.85	120	76.2	73.3	77.0	103.9	77.2	<0.07	<0.06	NA	<94.56	<107.68	Low	NA	60.1	46.7	67.9	84.6	65.7
**TBBL-0000317**	94.96	90.38	90.44	79.32	120	13.3	2.4	31.5	13.4	17.4								NA, low PS	NA, low PS	NA, low PS	NA, low PS	NA, low PS
**TBBL-0000468**	83.23	56.36	101.42	49.27	120	72.0	80.8	77.2	101.7	73.6	<0.25	<0.21	NA	<102.74	<109.10	Low	NA	21.9	7.3	1.7	39.1	21.1
**TBBL-0000552**	110.91	105.98	104.8	61.46	120	89.4	77.8	77.9	118.9	78.6	<0.43	0.38	NA	<105.81	122.87	Low	NA	80.2	58.3	56.1	89.1	85.7
**TBBL-0000539**	??	??	104.45	90.77	120	92.4	98.2	79.7	120.8	83.4	<0.10	0.31	>3.03	<96.40	105.53	Low	Likely	84.3	5.2	20.9	61.3	56.9
**TBBL-0000306**	102.19	98.38	110.19	92.52	120	33.8	23.0	16.6	2.0	9.5								NA, low PS	NA, low PS	NA, low PS	NA, low PS	NA, low PS
**TBBL-0000419**	96.78	97.63	82.68	0.55	120	78.2	45.6	78.9	96.2	75.6	0.18	0.32	1.79	95.21	102.48	Low	Poor or no	70.7	89.3	81.5	76.2	73.6
**TBBL-0000655**	85.48	62.01	88.17	46.25	120	76.7	81.8	71.5	58.2	80.3	<0.10	<0.09	NA	<98.59	<104.05	Low	NA	0.0	9.4	22.0	52.3	16.7
**TBBL-0000651**	108.57	82.62	107.38??	170??	120	86.7	89.4	61.7	91.1	55.8	<0.38	<0.35	NA	<80.07	<111.06	Low	NA	0.0	0.0	0.0	0.0	15.8
**TBBL-0000660**	88.37	85.07	86.17	10.78	120	83.9	85.7	52.5	92.0	63.1								15.3	4.3	18.1	26.6	7.7

NA, not applicable
NA, low PS, not applicable due to low plasma stability

Table S6. Pharmacokinetics of β-lactams in uninfected CD-1 mice.

		i.v. dosing (5 mg/kg)					p.o. dosing (25 mg/kg)	
**Origin**	**TBBL #**	**$AUC_{[0-5h]}$ (ng*h/mL)**	**Half-life (h)**	**k_{el} (h^{-1})**	**Vd (L/kg)**	**Clearance (mL/(kg*h))**	**$AUC_{[0-5h]}$ (ng*h/mL)**	**Bioavailability (%)**
GSK	TBBL-0000001	5588	0.50	1.40	0.32	444	111	0.19
GSK	TBBL-0000004	5075	0.54	0.96	1.02	985	n.d	n.d.
GSK	TBBL-0000005	3330	0.54	1.28	0.23	300	97	0.11
GSK	TBBL-0000009	20334	0.50	1.40	0.18	255	1356	1.33
GSK	TBBL-0000012	15212	0.38	1.81	0.18	330	559	0.73
Sanofi	TBBL-0000310	39113	0.48	1.45	0.09	128	614	0.34
Sanofi	TBBL-0000316	37128	0.58	1.20	0.11	135	542	0.30
Lilly	TBBL-0000552	7573	0.78	0.89	0.74	660	42	0.11

i.v.: intravenous; p.o.: oral; **$AUC_{[0-5h]}$:** area under the concentration-time curve from 0 to 5h post-dose; **k_{el}**: elimination rate constant; **V_d**: volume of distribution. **n.d.**, not determined.

Table S7. Pharmacokinetics after subcutaneous dosing of uninfected DHP-1 mice.

Compound	Dose (mg/kg)	C_{max}	T_{max} (h)	AUC_{last} (h.ng/mL)	T_{last} (h)	Comments
TBBL-0000001	300	29750 ± 7142	1.0	43025 ± 1685	2.0 – 4.0	Recovered at t= 8h
TBBL-0000005	50	26600 ± 10037	0.25 – 0.5	18151 ± 7177	2.0 – 4.0	No side effects
TBBL-0000009	300	2647 ± 266	2.0 – 4.0	8811 ± 675	4.0	ł
TBBL-0000316	300	28700 ± 866	1.0 – 2.0	60215 ± 11370	2.0 – 4.0	ł
TBBL-0000552	300	29900 ± 11001	1.0 – 2.0	61949 ± 24926	4.0	ł
TBBL-0000652	300	22233 ± 21724	1.0 – 2.0	29369 ± 32858	2.0 – 4.0	Recovered at t= 8h

ł, mice sacrificed.; **Cmax**: Maximum observed concentration; **Tmax**: Time to reach **Cmax**; **AUC_{last}**: Area under the concentration-time curve from the time of dosing to the time of the last quantifiable concentration (**T_{last}**); Mean of parameter ± standard deviation (n=3), Tmax and Tlast reported as a range.

Table S8. Pharmacokinetics of β-lactams in Mtb-infected DHP-1 knockout mice after subcutaneous administration.

Compound	Dose (mg/Kg)	C_{max} (ng/mL)	T_{max} (h)	AUC_{last} (h*ng/mL)	T_{last} (h)
Meropenem	50	724	0.5	387	1.0
Meropenem	300	3340	0.5	1998	1.0
Cefdinir	50	8730	0.5	4628	1.0
TBBL-0000001	50	27800	1	38126	3.0
TBBL-0000005	50	4130	0.5	7924	6.0
TBBL-0000009	50	380	0.5	975	6.0
TBBL-0000316	50	17500	0.5	23721	6.0
TBBL-0000552	50	6190	0.5	3658	3.0
TBBL-0000652	50	8320	1	11486	3.0

Cmax: Maximum observed concentration; **Tmax**: Time to reach **Cmax**; **AUC_{last}**: Area under the concentration-time curve from the time of dosing to the time of the last quantifiable concentration (**T_{last}**)

Table S9. Enumeration of viable *Mycobacterium tuberculosis* from lungs of untreated DHP-1 knockout mice or DHP-1 knockout mice treated with test β-lactams and detailed description of mouse health and viability.

	Log$_{10}$ CFU per mouse (lungs)						
	**Mouse 1**	**Mouse 2**	**Mouse 3**	**Mouse 4**	**Mouse 5**	**Mean**	**SD**
Untreated mice (Day 9)	7.9	7.0	7.4	7.3	7.5	**7.4**	**0.3**
Untreated mice (Day 16)	9.2	9.3	9.1	8.9	8.8	**9.1**	**0.2**
MRP + CLV (300+100 mg/kg)	7.3	7.1	7.3	7.0	7.0	**7.1**	**0.1**
Cefdinir + CLV	8.3[1]	8.8	8.4	8.6	8.4	**8.5**	**0.2**
MRP + CLV (50+100 mg/kg) [a]	7.4	7.5	8.1	7.8	7.3	**7.6**	**0.3**
TBBL-0000001 + CLV	8.9	8.7	8.7[1]	8.5[1]	9.0	**8.8**	**0.2**
TBBL-0000005 +CLV	9.0	8.7	8.9	8.8	8.9	**8.9**	**0.1**
TBBL-0000009 + CLV	8.7[1]	8.7[1]	8.5[1]	8.8	8.8[2]	**8.7**	**0.1**
TBBL-0000316 + CLV	8.0	8.1[1]	8.5	8.1	8.2[1]	**8.2**	**0.2**
TBBL-0000552 + CLV	9.1	9.0[1]	9.8	9.5	9.4	**9.4**	**0.3**
TBBL-0000652 + CLV	8.6	8.8	6.4[3]	8.5[1]	8.6	**8.6**	**0.1**

1: These mice met the withdrawal criteria (weight loss >20%) one day before the end of the experiment (on day 15) and were sacrificed. These mice were included in the analysis because they had received all doses of compounds.

2: This mouse showed bad clinical status and was close to 20% of weight loss. It was humanely sacrificed on day 15.

3: This mouse was considered an outlier due to CFU values being over two orders of magnitude different than the other mice in its group.

[a] Statistical comparison of meropenem 50 mg/kg + clavulanate versus other control and test beta-lactams:

- MERO 50 mg/kg + CLV vs 316 + CLV: $p<0.01$
- MERO 50 mg/kg + CLV vs any of the rest of compounds + CLV: $p<0.0001$ (including Cefdinir + CLV and others)

Clavulanate (CLV), po, bid, 100 mg/kg
Meropenem (MRP), sc, bid, 50 and 300 mg/kg
Rest of products, sc, bid, 50 mg/kg

REFERENCES

1. Lopez Quezada, L.; Li, K.; McDonald, S. L.; Nguyen, Q.; Perkowski, A. J.; Pharr, C. W.; Gold, B.; Roberts, J.; McAulay, K.; Saito, K.; Somersan Karakaya, S.; Javidnia, P. E.; Porras de Francisco, E.; Amieva, M. M.; Di Az, S. P.; Mendoza Losana, A.; Zimmerman, M.; Liang, H. H.; Zhang, J.; Dartois, V.; Sans, S.; Lagrange, S.; Goullieux, L.; Roubert, C.; Nathan, C.; Aube, J., Dual-Pharmacophore Pyrithione-Containing Cephalosporins Kill Both Replicating and Nonreplicating *Mycobacterium tuberculosis*. *ACS Infect Dis* **2019,** *5* (8), 1433-1445. DOI: 10.1021/acsinfecdis.9b00112.

2. Gold, B.; Roberts, J.; Ling, Y.; Quezada, L. L.; Glasheen, J.; Ballinger, E.; Somersan-Karakaya, S.; Warrier, T.; Warren, J. D.; Nathan, C., Rapid, Semiquantitative Assay To Discriminate among Compounds with Activity against Replicating or Nonreplicating *Mycobacterium tuberculosis*. *Antimicrob Agents Chemother* **2015,** *59* (10), 6521-38. DOI: 10.1128/AAC.00803-15.
